# Genotype by environment interactions for reproductive performance of North American purebred sows between North America and Southeast Asia

**DOI:** 10.1093/jas/skaf191

**Published:** 2025-06-19

**Authors:** Huy N P Pham, Robert A Kemp, Anna Wolc, Jack C M Dekkers

**Affiliations:** Department of Animal Science, Iowa State University, Ames IA 50011, USA; RAK Genetic Consulting Ltd, Lethbridge, ABCanadaT1K 6V1; Hy-Line International, Dallas Center, IA 50063, USA; Department of Animal Science, Iowa State University, Ames IA 50011, USA

**Keywords:** genotype-by-environment interactions, purebred sows, reproductive performance, swine

## Abstract

Importing improved Western pig genetics into Southeast Asia has been a common practice to enhance the reproductive performance of pork production in the region. This study aimed to investigate the presence and magnitude of genotype-by-environment (GxE) interactions for sow reproductive performance of purebred North American genetics between temperate (North America) and tropical climates (Southeast Asia). Reproductive data from North American purebred Landrace (LR) and Large White (LW) sows, raised in Canada and in 2 Southeast Asia nucleus herds were used to estimate genetic parameters and quantify GxE. Data were recorded from 2015 to 2023 for 6 reproductive traits: total number born (TNB), number born alive (NBA), number stillborn (NSB), number mummified, age at first farrowing (AFF), and farrowing interval (FI). On average, TNB and NBA were lower in Southeast Asia than in Canada for both LR (by 13.0%) and LW (by 11.1%). The Canadian data showed higher estimates of heritability and repeatability than the Southeast Asia data for TNB, NBA, and NSB. Estimates of genetic correlations between parities for TNB, NBA, and NSB were not significantly different from 1 in Southeast Asia for both LR and LW, but they were significantly different from 1 for both breeds in Canada. Estimates of genetic correlations between Canada and Southeast Asia for TNB, NBA, and NSB were significantly different from 1 for the LW breed, ranging from 0.54 to 0.66, but were higher for the LR breed, ranging from 0.81 to 0.92, and not significantly different from 1. Estimates of genetic correlations between the 2 Southeast Asian herds; however, also revealed the potential presence of GxE within Southeast Asia, although these estimates were associated with high standard errors and were not significantly different from 1. Estimates of the genetic correlation between regions for FI and AFF were found to differ between breeds, with LR showing negative genetic correlations (−0.10 ± 0.33 and −0.59 ± 0.29 for FI and AFF, respectively), while LW showed positive genetic correlations for these 2 traits (0.73 ± 0.41 and 0.50 ± 0.11, respectively). The higher estimates of genetic correlations for reproductive traits between Canada and Southeast Asia for the LR breed indicate that LR sows may be more robust when exposed to a tropical climate, although there was no difference between the 2 breeds in the drop in average reproductive performance between Canada and Southeast Asia, nor was a seasonal effect on performance within Canada and Southeast Asia more pronounced for LW than LR. Further research is needed to investigate the differences in robustness and adaptability between these 2 breeds.

## Teaser

This research reveals critical genotype-by-environment interactions affecting the reproductive performance of North American purebred sows and suggests that Landrace sows may be more robust when exposed to tropical climates than Large White sows.

## Introduction

To meet the demand for animal-sourced protein in the region, many farmers in Asia have imported improved genetics from North America and Europe since the early 19th century, with the aim to enhance the reproductive traits and productivity of pork production in the region (Trovão and Nelson, 2020). However, the effectiveness of this approach is not clear, considering the diverse environmental conditions between countries and herds, such as health status, feed quality, on-farm management, and climate conditions (FAO, 2019). Diverse climatic environments may reduce the accuracy of estimated breeding values (EBV) for a new environment if genotype-by-environment (GxE) interactions are not accounted for in genetic evaluation (Knap and Su, 2008; Nirea and Meuwissen, 2017). Thus, the ranking of animals based on their EBV could differ between environments due to GxE, meaning that animals selected based on EBV for a certain environment may not be the best-expected performers under divergent environmental conditions (Banos and Smith, 1991).

In livestock breeding, GxE can be analyzed by treating phenotypes of the same trait that are recorded under different environmental conditions as distinct yet genetically correlated traits and analysis using a multi-trait model. In this case, a deviation of the genetic correlation from one indicates the presence of GxE (Falconer, 1952). Robertson (1959) suggested that GxE is of biological and agricultural importance if the genetic correlation for the same trait in different environments is below 0.80. This multi-trait approach is extensively applied when contrasting specific environmental conditions, including climate, e.g., temperate versus tropical (Mote et al., 2000) , mating methods, e.g., artificial insemination compared to natural service (Lewis and Wiseman, 2006), and different rearing systems, e.g., organic versus conventional farming (Wallenbeck et al., 2008). 

Reproductive performance is crucial in any swine breeding program (Rothschild and Ruvinsky, 2011) and selection for reproduction traits has shown great progress in the past decades (Knap et al., 2023). However, climate conditions pose significant constraints on pig reproductive performance and, with current trends in moving pig production to warmer climates and global warming, maximizing reproductive efficiency across different environments is becoming increasingly crucial. Pigs are sensitive to changes in ambient temperature due to their physiological characteristics of self-thermo regulation. Even with optimal within-barn environmental management strategies, pigs can still be affected by daily weather changes (Tiezzi et al., 2020). As such, when temperatures within the barn rise beyond the pigs’ optimal thermal zone, they experience heat stress, which can lead to reductions in feed intake, milk production (Cervantes et al., 2018; Serviento et al., 2020), animal welfare (Johnson et al., 2019), and reproductive performance, such as increasing the incidence of anestrous and embryonic death (Ross et al., 2015), a decrease in litter size traits (Suriyasomboon et al., 2006), an increase in the number of stillborn piglets (Wegner et al., 2016), and a decrease in boar sperm mobility and fertility (Wettemann et al., 1976). In the United States alone, heat stress is estimated to result in a total economic loss of approximately $450 million annually for the swine industry (Pollmann, 2010).

The main objective of this study was to investigate the magnitude of GxE for the reproductive performance of North American purebreds under temperate (Canada) and tropical (Southeast Asia) climatic environments, hypothesizing that estimates of genetic correlations for reproductive traits between North American and Southeast Asian herds are significantly different from 1. To accomplish this, analyses conducted included 1) estimating genetic parameters of North American purebred sows reproduction traits within temperate (Canada) and tropical (Southeast Asia) climatic environments and 2) quantifying the presence of GxE for sow reproductive traits between North American and Southeast Asian herds.

## Materials and Methods

### Climate data

Reproduction records were obtained from sows that were housed in Southern Manitoba, Canada, and in 2 locations in Southeast Asia. To characterize the climates at these 3 locations, meteorological data were extracted from the Meteoblue (Meteoblue History Plus) Historical Data database for Southern Manitoba and for the 2 herd locations in Southeast Asia, consisting of daily average, maximum, minimum temperatures (°C), and relative humidity (%)during the period over which records were available, from 2015 to 2023. The daily temperature-humidity index (THI) was calculated using the formula proposed by NWSCR (National Weather Service Central Region (NWSCR), 1976):


$$\begin{array}{l} THI=\left(1.8\;T+32\right)-\left(0.55\left(\frac{RH}{100}\right)\right)\left(\left(1.8\;T+32\right)-58\right)\; \end{array}$$ (1)

where T is the observed temperature in degree Celsius (°C) and RH is the observed relative humidity on a 0 to 100 scale.

### Phenotypes and pedigree

An overview of the available reproduction data is given in Table 1. Genesus (Oakville, Canada) provided reproduction data from purebred Landrace (LR) and Large White (LW) sows from their nucleus herds in Canada (CA) and Southeast Asia (AS). All matings were by artificial insemination. The Canadian dataset included 77,271 farrowing records from 10,345 LR sows and 17,695 LW sows that farrowed from 2015 to 2023 in 7 nucleus herds in southern Manitoba under temperate climate conditions. All barns are closed-housing systems with controlled lighting and ventilation, while nutrition specifications and management follow central management guidelines and are slightly modified depending on each barn’s facilities. The AS dataset included 17,569 farrowing records from 3,739 LW sows and 3,193 LR sows that farrowed from 2020 to 2023 in 2 nucleus herds under tropical climate conditions. The 2 Asian herds were initially established by importing gilts from the Genesus purebred lines located in southern Manitoba. These purebred gilts were then reared under the closed-housing system in Tay Ninh province, Vietnam, and on Luzon Island, Philippines. Both herds were provided with the same nutritional specifications, management guidelines, and on-site technical support used in Canada. Subsequently, these herds received additional germplasm from the Canadian lines by importing semen. Details regarding the total number of gilts, the number of imported gilts, and the number of contemporary groups with imported gilts are provided in Table 2.

**Table 1.** Number of litter records and sows for each breed in Canada and 2 herds in South-East Asia^1^

Numbers of	Breed	Region		
		Canada	Asia 1	Asia 2
Litters	Large white	51,375	7,861	2,082
	Landrace	25,896	4,061	3,565
Sows	Large white	17,695	2,753	986
	Landrace	10,345	1,717	1,476

^1^There were 7 herds for the Canadian dataset. Four of the herds contained only Large White.

**Table 2.** Number of imported gilts in the 2 Southeast Asian herds

	Landrace		Large white	
	Asia 1	Asia 2	Asia 1	Asia 2
Total number of gilts	1,691	1,283	2,616	830
Number of imported gilts	213	399	1,513	210
Total number of CG	28	20	36	21
Number of CG with imported gilts	5	4	8	4
Time period of gilt imports	June 2020 to October 2020	July 2020 to October 2020	May 2020 to December 2020	July 2020 to October 2020

CG, Contemporary group.

For each farrowing record, sow identification, birth date, farrowing date, parity, farm, sow breed, service sire identification, and service sire breed were available, as documented by herd staff. Reproductive traits available for each farrowing record included total number born (TNB), number born alive (NBA), number of mummified piglets (NM), number stillborn piglets (NSB), farrowing interval (FI), and age at first farrowing (AFF). NBA is the number of piglets observed alive in a litter within 24 h of birth. NSB is the number of piglets that were observed dead within 24 h of birth, excluding NM. TNB was calculated as NBA plus NSB. AFF was computed as the age of the sow at her first farrowing, while FI was computed as the number of days between 2 consecutive farrowing dates of the sow. In CA, farrowing records were recorded as soon as the sow finished farrowing or as soon as the litter was observed after the sow had finished farrowing. In AS, farrowing records were recorded on day 1 after farrowing. Pedigree data for the LR and LW sows included 16,140 and 26,115 animals, respectively, with over 15 generations for the Canadian dataset and over 10 generations for the Asian dataset.

### Data editing

Data editing was performed using R Studio (Team, 2021). Duplicate farrowing records were removed and animals with incomplete reproductive information, including unknown service sire breeds or those appearing in more than one herd, were also excluded. Phenotyped animals that were not included in the pedigree were excluded from the dataset. Records with a FI exceeding 200 d were also excluded from the dataset. Records lacking a service sire identification were assigned a unique service sire number for each farrowing record, noting that service sire breed information was available for all of these records. In both the CA and AS datasets, sows with a recorded AFF greater than 420 d were adjusted from parity 1 to parity 2, assuming the first parity was not recorded, and similar adjustments were made for subsequent parities of these sows, where applicable. In total, the parity number was modified for 2,143 records and 1,146 records were deleted. The number of records means, and standard deviations of the traits analyzed in the edited data set are given in Table 3.

**Table 3.** Descriptive statistic for reproductive traits for each breed in each region

Trait^1^/ region^2^	Number of records			Mean			Standard deviation^3^		
	CA	AS1	AS2	CA	AS1	AS2	CA	AS1	AS2
Landrace									
TNB	25,896	4,061	3,565	14.45	12.18	12.74	3.22	3.48	3.28
NBA	25,896	4,061	3,565	13.16	11.70	11.99	3.07	3.56	3.30
NM	25,896	4,061	3,565	0.51	0.51	0.64	0.88	1.28	1.01
NSB	25,896	4,061	3,565	1.30	0.48	0.75	1.57	1.19	0.88
AFF	9,996	1,691	1,283	357.30	331.89	362.81	18.41	15.20	15.09
FI	14,984	2,300	2,063	147.80	150.68	151.31	8.97	11.24	10.25
Large white									
TNB	51,375	7,861	2,082	15.38	13.49	13.52	3.86	3.54	4.02
NBA	51,375	7,861	2,082	13.59	12.45	12.37	3.62	3.92	4.02
NM	51,375	7,861	2,082	0.53	0.54	0.70	1.03	1.06	1.39
NSB	51,375	7,861	2,082	1.79	1.04	1.15	1.91	1.71	1.82
AFF	17,632	2,617	830	356.0	341.16	364.11	23.80	15.20	14.68
FI	32,971	4,760	814	145.6	148.32	150.70	10.36	10.53	10.35

^1^Reproductive traits: total number born (TNB), number born alive (NBA), number mummified (NM), number stillborn (NSB), age at first farrowing (AFF), farrowing interval (FI).

^2^Regions: Canadian herds (CA), Southeast Asian herd 1 (AS1), Southeast Asian herd 2 (AS2).

^3^Standard deviation was calculated from phenotypic variance from the univariate model.

### Genetic connectedness

To ensure the reliability of the results and evaluate genetic relationships between sows in the CA and AS herds, genetic connectedness between phenotyped animals was evaluated using the additive genetic relationship matrix (A-matrix) method, as proposed by Banos & Cady (1988). The pedigree-based relationship matrix for a given breed (LR or LW) was partitioned as follows:


$$\begin{array}{l} A=\;\left[\begin{array}{lllllll} A_{CA} & A_{CA,AS1} & A_{CA,AS2}\;A_{AS1,CA} & A_{AS1} & A_{AS1,AS2}\;A_{AS2,CA} & A_{AS2,AS1} & A_{AS2}\; \end{array}\right]\; \end{array}$$ (2)

where subscripts CA, AS1, and AS2 refer to blocks of relationships involving animals phenotyped in Canada, Southeast Asian herd 1, and Southeast Asian herd 2, respectively. The average additive relationship was calculated within and between each group of animals (CA, AS1, and AS2), ignoring diagonal elements for diagonal blocks. Additive genetic relationships between phenotyped animals were also visualized using heatmaps, separately for LR and LW. For this purpose, the relationship matrix in [2] was ordered by region (CA, AS1, AS2) and by date of birth of the phenotyped animals (oldest to youngest). Heatmaps were generated using the ggplot2 package in R (Wilkinson, 2011). In addition, principal component analysis of the A-matrix from the phenotyped animals was performed using the prcomp function in R (R Core Team, 2024) to further assess population structure and genetic relationships between CA, AS1, and AS2.

### Genetic parameters by region

All (co)variance components were estimated using the ASREML software version 4.2 (Gilmour, A.R. et al., 2021) based on the models described in the following sections.

Genetic parameters were estimated using restricted maximum likelihood (REML) analyses based on the following general multi-trait animal model, separately for each breed:


$$\begin{array}{l} y=Xb+Za+W_{1}ss+W_{2}pe+\;e \end{array}$$ (3)

where **y** is the vector of phenotypic records of sows; **b** is the vector of fixed effects; **a** is the vector of additive genetic values of sows; **ss** is a vector of service sire effects; **pe** is a vector of permanent environmental effects of sows; **e** is a vector of random residuals; and **X**, **Z**, **W**_**1**,_ and **W**_**2**_ are the corresponding incidence matrices. In general, for TNB, NBA, NSB, NM, and FI, the fixed effects included contemporary group, service sire breed, and parity. For AFF, the model included only contemporary groups and service sire breeds as fixed effects. For both CA and AS, the fixed effect of parity was fitted with 3 classes to ensure a sufficient number of records for each parity class, i.e., Parity 1, Parity 2, and Parity ≥3. For FI, parity was defined as the parity class of the litter that followed the interval. The contemporary group was defined as herd-week-year of farrowing (HWY) for the CA data but as herd-month-year of farrowing (HMY) for the AS data, to ensure an adequate number of observations within each contemporary group. The effect of crossbred versus purebred litters was captured by fitting service sire breed as a fixed effect, i.e., LR or LW. For the AS data, we also included the classification of the sow having been imported or not as a fixed effect to account for potential differences between these groups.

For multivariate versions of the model [3], distributional assumptions for random effects were:


$$\begin{array}{l} \left[\begin{array}{c} a\;ss\;pe\;e \end{array}\right]∼N\left(\left[\begin{array}{c} 0\;0\;0 \end{array}\right]\begin{array}{c} ,\left[\begin{array}{ccccccccccccc} G⊗A & 0 & 0 & 0\;0 & SS⊗I & 0 & 0\;0 & 0 & PE⊗I & 0\;0 & 0 & 0 & R⊗I \end{array}\right] \end{array}\right) \end{array}$$ (4)

where ***G*** is the additive genetic (co)variance matrix between traits; ***A*** is the relationship matrix, as in equation [2]; ***SS*** is the service sire (co)variance matrix; ***I*** is an identity matrix; ***PE*** is the permanent environmental (co)variance matrix; and ***R*** is the residual (co)variance matrix.

Variance components for each trait were estimated using the average-information algorithm applied to the univariate version of the model [3], i.e., additive genetic variance $\left(\sigma_{a}^{2}\right)$, service sire variance $\left(\sigma_{ss}^{2}\right)$, permanent environmental variance $\left(\sigma_{pe}^{2}\right)$, and residual variance $\left(\sigma_{e}^{2}\right)$. Subsequently, estimates of heritability ($h^{2}$), repeatability (*rpt*), and the proportion of phenotypic variance explained by service sire (*ss*) were calculated based on the following functions:


$$\begin{array}{l} h^{2}=\frac{\sigma_{a}^{2}}{\sigma_{P}^{2}};rpt=\frac{\sigma_{a}^{2}+\sigma_{pe}^{2}}{\sigma_{P}^{2}};ss=\frac{\sigma_{ss}^{2}}{\sigma_{P}^{2}}\; \end{array}$$ (5)

where $\sigma_{P}^{2}$ is the phenotypic variance, calculated as the sum of $\sigma_{a}^{2}$$,\sigma_{ss}^{2}$, $\;\sigma_{pe}^{2}$ and $\sigma_{e}^{2}$ for TNB, NBA, NM, NSB, FI, and as the sum of $\sigma_{a}^{2}$, $\sigma_{ss}^{2},\;and\;\sigma_{e}^{2}$ for AFF. Phenotypic $(r_{p_{x,y}})\;$ and genetic $(r_{g_{x,y}})\;correlations\;\;\;$ between traits were estimated using bivariate versions of the model [3] and computed using estimates of variances and covariances as:


$$\begin{array}{l} r_{g_{x,y}}=\frac{cov(a_{x},a_{y})}{\sigma_{a_{x}}\sigma_{a_{y}}};r_{p_{x,y}}=\frac{cov(p_{x},p_{y})}{\sigma_{p_{x}}\sigma_{p_{y}}} \end{array}$$ (6)

where $a_{x}$ and $a_{y}$ represent the additive genetic effects of traits × and trait y for the same individual and $p_{x}$ and $p_{y}$ represent the phenotypes for traits × and trait y for the same individual.

The multivariate version of the model [3] was used to estimate genetic and phenotypic correlations for each trait between parities for TNB, NBA, NSB, and NM. Four parities (Parity 1 to 4) were assessed in the CA dataset. The multivariate models for the CA dataset included only additive genetic effects as a random effect due to convergence problems. Due to limited size, we were unable to perform multivariate analysis on 4 separate parities in the AS datasets. Therefore, we grouped the parities into 3 classes: Parity 1, Parity 2, and Parity ≥3. For all 3 classes, additive genetics was fitted as a random effect, along with a permanent environmental effect for Parity ≥3, to account for repeated records.

Prompted by the initially observed high estimate of heritability for AFF for LW in AS1, which was suspected to be the result of different treatment of imported gilts, the AFF data of gilts in as the AS datasets were also analyzed using a bivariate model version of the model [3] that assumed AFF was a different trait for imported versus non-imported gilts. Fixed effects included the service sire breeds and contemporary group. Random effects included animal additive genetic effects and service sire effects. Environmental covariances between these 2 traits were not estimable and were set to zero.

### Genotype by environment interactions

GxE was quantified by the method proposed by Falconer (1952) by using multi-trait animal models, separately for each of the 6 evaluated traits, by considering the phenotype in each region as a separate but correlated trait. Traits from different populations were combined pairwise, including CA with AS1 (CA-AS1), CA with AS2 (CA-AS2), CA with the combined AS data (CA-AS), and AS1 with AS2 (AS1-AS2). The bivariate version of the model [3] was then used to estimate (co)variance components for each pair of populations. Relationships among animals, both within and between populations, were included using a pedigree-based additive genetic relationship matrix. Since only a limited number of service sires were used across regions, the covariance of service sire effects between regions was set to zero. Also, no animal had records in different regions, thus, the covariances of permanent environmental effects between regions were not estimable and were also set to zero.

### Significance of correlation estimates

Z-tests were conducted to compare estimates of genetic and phenotypic correlations between regions *i* and *j* for a given pair of traits, as follows (Howell, 1992):


$$\begin{array}{lll} Z_{i} & \;=\frac{1}{2}\log\left(\frac{1+r_{i}}{1-r_{i}}\right);n_{i}=\frac{1-r_{i}}{SE_{r_{i}}^{2}}+2;s\; & \;=\sqrt{\frac{1}{n_{i}-3}+\frac{1}{n_{j}-3}};Z_{score_{ij}}\;=\frac{Z_{i}-Z_{j}}{s} \end{array}$$ (7)

where $Z_{i}$ is the Fisher-z-transformation for the genetic or phenotypic correlation in each region; $r_{i}$ is the genetic or phenotypic correlation estimate; $SE(r_{i})$ is the estimated standard error for each genetic or phenotypic correlation; $n_{i},n_{j}$ are the sample sizes for each region; s is the standard error for the difference between correlation estimates. In addition, 95% confidence intervals of genetic correlation estimates were used to test whether estimates of the genetic correlation of reproductive traits between parities and between regions were significantly different from 1 at *P* < 0.05.

## Results

### Climate and average reproductive performance by region

**Figure 1.**
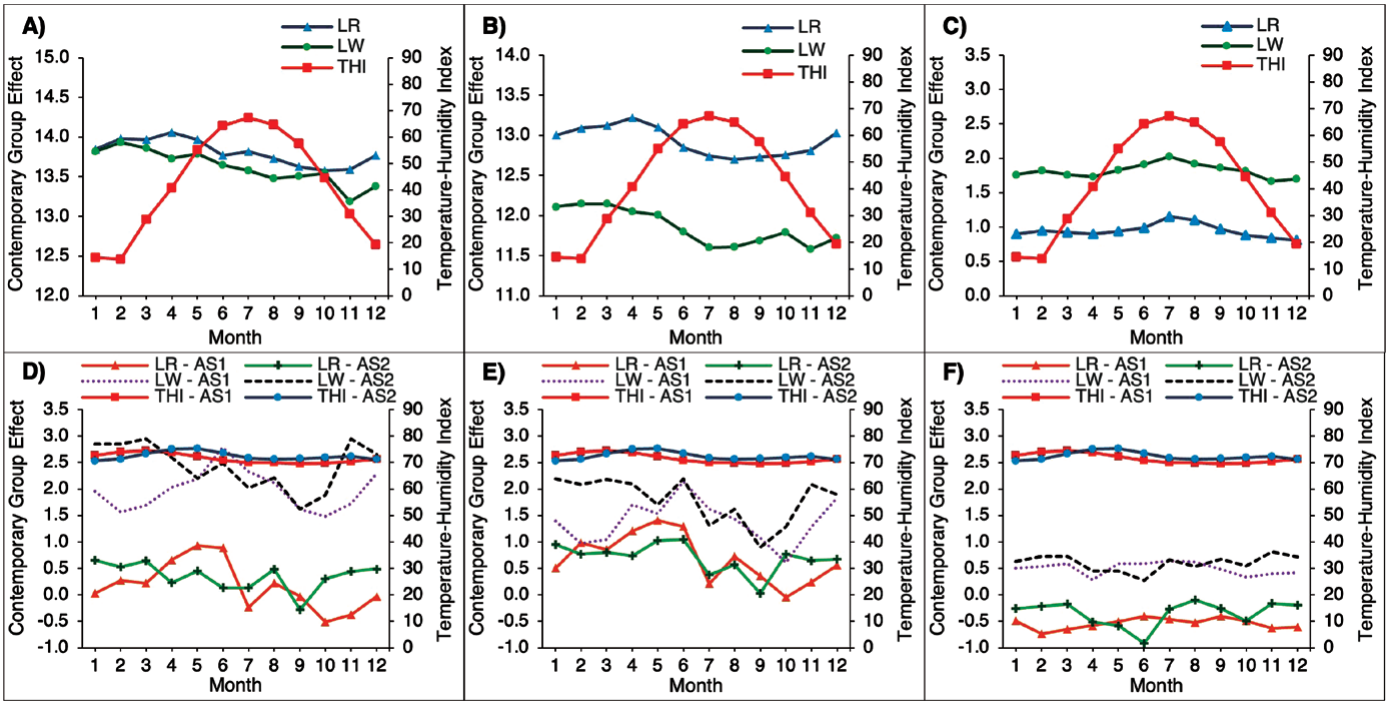
Monthly THI averaged over the years 2015 to 2023 and average estimates of corresponding contemporary group effects in Canada CA) (A, B, C) and 2 herds in Southeast Asia (AS1, AS2) (D, E, F) for the Landrace (LR) and Large White (LW) breeds. Contemporary group effects are for the total number born (A, D), number born alive (B, E), and number stillborn (C, F).

Figure 1 illustrates the THI by month, averaged across the years 2015 to 2023, for CA, AS1, and AS2, along with trends in the estimate of average contemporary effects obtained from the univariate model analyses of the reproduction traits. In AS, the average THI were consistently high (THI > 70) throughout the year. In contrast, the average THI varied seasonally in CA: low from December to February, moderate from March to May and from September to November, and high from June to August. These seasonal effects for THI in CA aligned with seasonal trends in the average of estimates of corresponding contemporary group effects, which are also shown in Figure 1. For TNB and NBA in CA (Figure 1A, B), there was an increase in average contemporary group effect estimates from December to April, followed by a decline, while average contemporary group effects for NSB increased from April to August (Figure 1C). Trends in estimates of contemporary group effects for TNB, NBA, and NSB were found to be similar between LR and LW. In contrast, no clear trends in contemporary group effect estimates were observed for TNB, NBA, or NSB for either AS1 or AS2 for either breed (Figure 1D, E, F). To further understand climate differences, we also plotted the daily average and range of THI in AS and in CA for each year from 2015 to 2023 in Figure S1. In AS, there were minimal fluctuations in average daily THI and a smaller range of daily THI compared to CA. The constant high THI in AS indicates that sows in AS1 and AS2 are continuously exposed to elevated thermal conditions. This consistent exposure may mask any potential seasonal effects on reproductive performance, as the sows do not experience substantial changes in environmental conditions throughout the year. In contrast, the greater variability in the daily average and range of THI in CA suggests that sows are exposed to more thermal fluctuating conditions, contributing to the observed seasonal trends in contemporary group effects shown in Figure 1(A, B, C).

Descriptive statistics for all reproductive traits are in Table 3. Comparing means for TNB and NBA, the sows in CA performed better than those in AS, by 13.0% and 11.1% for LR and LW, respectively. The 2 AS herds showed greater variability for TNB and NBA for LR sows, but the CA data showed greater variability for NSB and AFF for both LR and LW, compared to the 2 AS herds. For LR, on average, differences in standard deviations between CA and AS were 37.9% and 19.6% for NSB and AFF, respectively. For LW, these differences were 8.2% and 47.4% for NSB and AFF, respectively. Notably, while the expectation was for NSB to be greater for the AS compared to the CA data, because of the differences in when NSB was recorded relative to farrowing (see Materials and Methods), the results showed a greater mean and greater variation of NSB in CA for both breeds. Averages for FI were similar for both breeds and for both regions.

### Genetic connectedness

**Figure 2.**
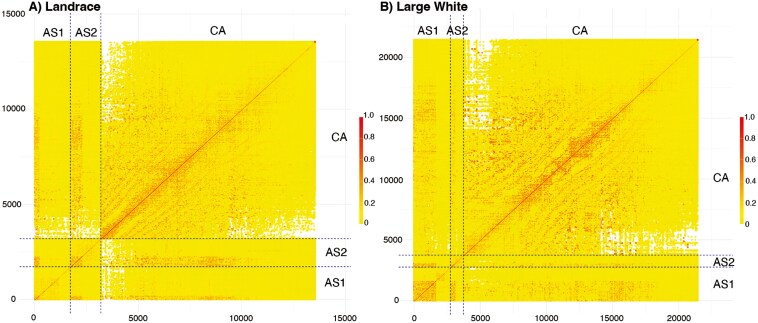
Heatmaps of the additive relationship matrix between phenotyped animals from Canada (CA), Southeast Asian herd 1 (AS1), and Southeast Asian herd 2 (AS2). A) Landrace breed, B) Large White breed. Captions: *Matrix was ordered by population and increasing date of birth. Dashed lines delineate cohorts AS1, AS2, and CA. Within each population, animals are arrayed from left to right and bottom to top from the oldest to the youngest based on their date of birth.

Genetic relationships between phenotyped animals within the LR and LW breeds within and between CA, AS1, and AS2 are illustrated in heat maps in Figure 2. Generally, there were genetic relationships between animals in CA and AS, but the strength of these relationships tended to decrease over time for both breeds. Average additive relationships within and between regions are shown in Table 4. For within-region relationships, in general, phenotyped sows in CA showed a lower average relationship compared to sows within AS1 and AS2 for both breeds. For both LR and LW, the average additive relationships of sows in CA with those in the 2 AS herds were generally lower than those observed within CA, AS1, and AS2. The average additive relationships of sows in CA with sows in each AS herd were about 50% lower than the average relationship between the 2 AS herds for both LR and LW.

**Table 4.** Average additive relationship within and between regions among phenotyped animals of 2 breeds (Landrace and Large White)

Breed	Landrace			Large white		
Submatrix	Matrix order	Average $A_{ij}$	SD	Matrix Order	Average $A_{ij}$	SD
Within region						
Canadian	10,345	0.0019	0.0714	17,695	0.0011	0.0710
Asian 1	1,717	0.0129	0.0724	2,753	0.0050	0.0890
Asian 2	1,476	0.0105	0.0861	986	0.0131	0.0870
Between regions						
Asian 1-Asian 2	1,717 × 1,476	0.0084	0.0861	2,753 × 986	0.0071	0.0590
Canadian—Asian 1	10,345 × 1,717	0.0038	0.0394	17,695 × 2,753	0.0022	0.0484
Canadian—Asian 2	10,345 × 1,476	0.0037	0.0532	17,695 × 986	0.0024	0.0388

SD, statistical standard deviation.

**Figure 3.**
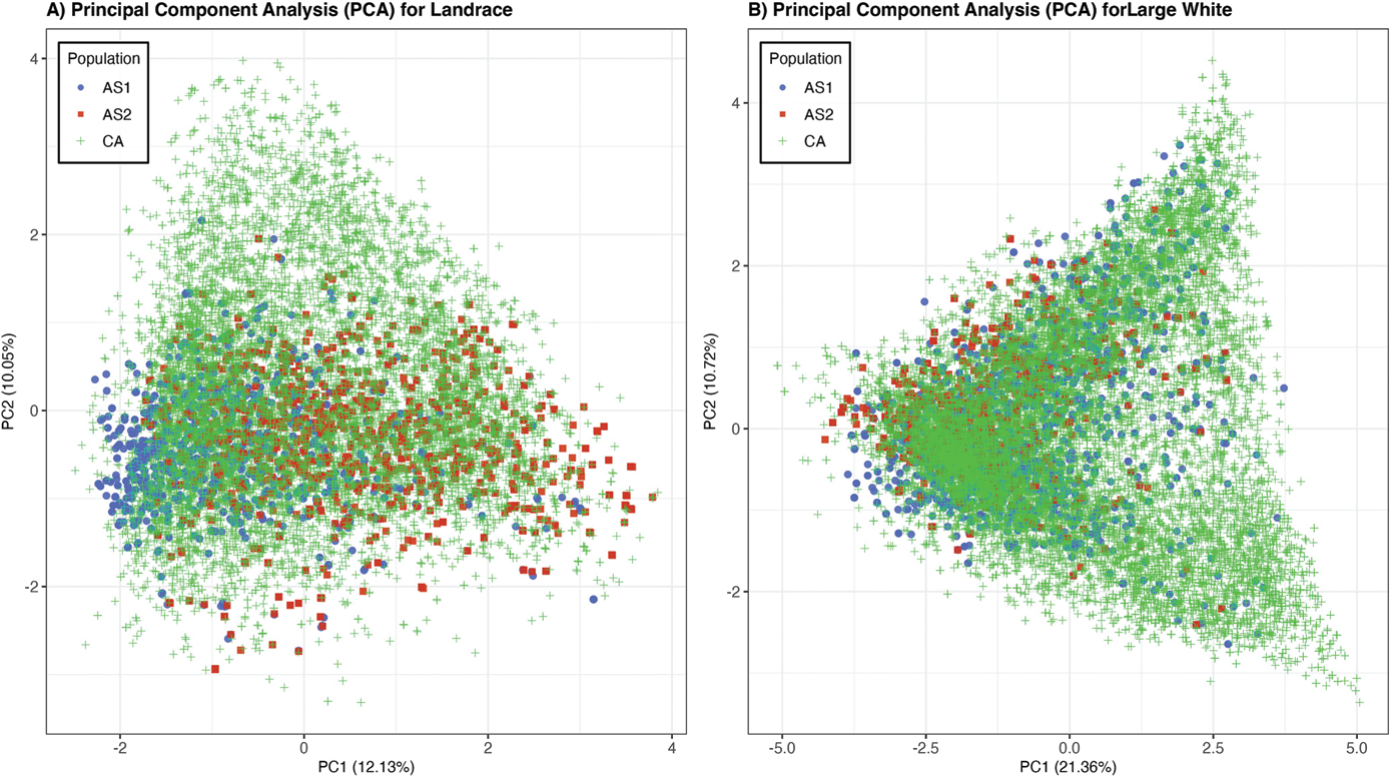
Principal component analysis (PCA) of pedigree-based relationships between phenotyped animals from Canada (CA), Southeast Asian herd 1 (AS1), and Southeast Asian herd 2 (AS2). A) Landrace breed, B) Large White breed.

Principal component analysis further illustrated the genetic structure of these populations (Figure 3). For LR (Figure 3A), the first 2 principal components explained 12.13% and 10.05% of the total variation, respectively. For LW (Figure 3B), PC1 and PC2 explained 21.36% and 10.72% of the total variation, with more pronounced population structure evidence compared to LR. Overall, for both breeds, the 3 populations overlapped well, which confirms the genetic connectedness between these populations.

### Univariate variance components and heritabilities

Based on the fitted univariate animal models, estimates of heritability, repeatability, and the proportion of phenotypic variance due to service sire, and their corresponding standard errors (SE) for the analyzed reproduction traits are in Table 5 for both breeds and regions. For both breeds, the CA data showed higher heritability estimates for TNB, NBA, and NSB than the AS data. For LR, estimates of heritability were 0.11 ± 0.01, 0.10 ± 0.01, and 0.12 ± 0.01 for TNB, NBA, and NSB, respectively, in the CA data, and 0.07 ± 0.02, 0.07 ± 0.01, and 0.06 ± 0.01 for TNB, NBA, and NSB, respectively, in the AS data. For LW, estimates of heritability were 0.07 ± 0.01, 0.07 ± 0.01, and 0.13 ± 0.01 for TNB, NBA, and NSB, respectively, in the CA data, and 0.05 ± 0.01, 0.06 ± 0.01, and 0.05 ± 0.01 for TNB, NBA, and NSB, respectively, in the AS data. For NM and FI, estimates of heritability were consistent across breeds and regions. For AFF, the LR breed also showed consistent heritability estimates between CA and AS (0.08 ± 0.01 for CA and 0.07 ± 0.01 for AS). For LW, estimates of heritability for AFF were inconsistent between CA and AS, with an estimate of 0.14 ± 0.01 in CA and 0.40 ± 0.04 in AS. Estimates of heritability for AFF were also different between AS1 and AS2 for LW, with a moderately high estimate for AS1 (0.45 ± 0.05) and a low estimate for AS2 (0.06 ± 0.05), while estimates for LR were low for both AS1 and AS2 (0.08 ± 0.03 and 0.09 ± 0.05, respectively).

**Table 5.** Estimates of heritability (h^2^), repeatability (rpt), and the proportion of phenotypic variance due to service sire (ss) for reproductive traits in the Landrace and Large White breeds for each region, with standard errors in parentheses

Breed	Landrace								Large White							
Trait^1^/Region^2^	CA		AS		AS1		AS2		CA		AS		AS1		AS2	
TNB																
$h^{2}$	0.11	(0.01)	0.07	(0.02)	0.03	(0.01)	0.12	(0.03)	0.07	(0.01)	0.05	(0.01)	0.06	(0.02)	0.04	(0.03)
rpt	0.20	(0.01)	0.12	(0.01)	0.10	(0.02)	0.13	(0.02)	0.18	(0.01)	0.14	(0.01)	0.13	(0.01)	0.15	(0.03)
ss	0.02	(0.00)	0.02	(0.01)	0.01	(0.01)	0.03	(0.01)	0.03	(0.00)	0.03	(0.01)	0.03	(0.01)	0.05	(0.02)
NBA																
$h^{2}$	0.10	(0.01)	0.07	(0.01)	0.03	(0.01)	0.12	(0.02)	0.07	(0.01)	0.06	(0.01)	0.06	(0.02)	0.05	(0.03)
rpt	0.18	(0.01)	0.11	(0.01)	0.10	(0.02)	-	-	0.17	(0.01)	0.15	(0.01)	0.14	(0.01)	0.17	(0.03)
ss	0.02	(0.00)	0.02	(0.01)	0.02	(0.01)	0.03	(0.01)	0.03	(0.00)	0.03	(0.01)	0.02	(0.01)	0.05	(0.02)
$h^{2}$	0.03	(0.01)	0.03	(0.01)	0.01	(0.01)	0.05	(0.02)	0.03	(0.00)	0.02	(0.01)	0.03	(0.01)	0.00	
rpt	0.05	(0.01)	0.04	(0.01)	-	-	0.06	(0.02)	0.05	(0.00)	0.03	(0.01)	-	-	0.05	(0.02)
ss	0.00	(0.00)	0.01	(0.00)	0.00	(0.01)	0.01	(0.00)	0.01	(0.00)	-	-	-	-	-	-
NSB																
$h^{2}$	0.12	(0.01)	0.06	(0.01)	0.03	(0.01)	0.08	(0.02)	0.13	(0.01)	0.05	(0.01)	0.05	(0.01)	0.02	(0.02)
rpt	0.20	(0.01)	0.08	(0.01)	-	-	0.11	(0.02)	0.18	(0.01)	0.06	(0.01)	0.07	(0.01)	0.06	(0.03)
ss	0.01	(0.00)	0.00	(0.00)	-	-	0.01	(0.00)	0.01	(0.00)	0.00	(0.00)	-	-	0.01	(0.01)
FI																
$h^{2}$	0.04	(0.01)	0.03	(0.02)	0.05	(0.02)	0.02	(0.02)	0.03	(0.01)	0.01	(0.01)	0.01	(0.01)	0.02	(0.04)
rpt .	0.07	(0.01)	0.07	(0.02)	0.11	(0.03)	-	-	0.05	(0.01)	0.02	(0.01)	0.02	(0.01)	0.06	(0.05)
ss	0.02	(0.01)	0.02	(0.01)	0.04	(0.02)	0.00	(0.01)	0.01	(0.00)	0.01	(0.02)	-	-	0.01	(0.01)
AFF																
$h^{2}$	0.08	(0.01)	0.07	(0.02)	0.08	(0.03)	0.09	(0.05)	0.14	(0.01)	**0.40** ^*^	(0.04)	**0.45** ^*^	(0.05)	0.06	(0.05)
rpt	-	-	-	-	-	-	-	-	-	-	-	-	-	-	-	-
ss	0.01	(0.00)	0.02	(0.01)	0.00	(0.02)	0.02	(0.01)	0.00	(0.00)	0.01	(0.01)	0.02	(0.02)	0.00	(0.01)

^1^Traits: TNB = total Number Born, NBA = number born alive, NSB = number of stillborn, NM = number of mummified, FI = farrowing interval, AFF = age at first farrowing.

^2^Regions: CA = Canada, AS = Southeast Asia, AS1 = Southeast Asian herd 1, AS2 = Southeast Asian herd 2.

^*^These estimates were biased upward by the apparent different management of imported gilts (see Supplementary Material for full details).

The high heritability estimate for AFF for LW in AS1 was further investigated by analyzing AFF of imported and non-imported gilts as separate traits, with results shown in Tables S2 and S3. The mean and SD of AFF for imported gilts for LW in AS1 were 350.3 and 21.3 days compared to 328.8 and 15.8 d for non-imported gilts. Due to problems with model convergence, the AFF of imported gilts were pre-corrected for the effect of CG by subtracting the difference between the average AFF of non-imported gilts within their CG and the average of all imported gilts. After implementing this adjustment, all imported gilts were assigned to a single CG. For non-imported gilts, the CG were kept as original. This assumes that the LW gilts in AS1 that were imported at different times did not substantially differ in average breeding value for AFF, which was plausible because of the limited number of CG for imported gilts (8, see Table 2) and the short timeframe over which they were born (from June to October). These analyses were not conducted for LW in AS2, nor for LR in either AS1 or AS2 or the combined dataset because of the small numbers of imported gilts for these populations. Results showed that, in AS1, AFF had a higher estimate of heritability for imported LW gilts (0.84 ± 0.08) than for non-imported LW gilts (0.19 ± 0.07). Estimates of additive genetic and phenotypic variances of AFF for LW in AS1 were 226.21 ± 31.95 and 266.54 ± 14.54, respectively, for imported gilts, and 37.25 ± 13.95 and 197.68 ± 9.41, respectively, for non-imported gilts. Moreover, the estimate of the genetic correlation for AFF between imported and non-imported gilts for LW in AS1 was relatively low (0.33 ± 0.21; Figure S4). Because AS2 had relatively fewer imported LW gilts, similar estimates of heritability were obtained in the combined AS data, with a higher estimate for imported gilts (0.81 ± 0.08) compared to non-imported gilts (0.07 ± 0.04) and a moderately positive estimate of the genetic correlation between imported and non-imported gilts (0.64 ± 0.28; Figure S4).

Estimates of the proportion of phenotypic variance in litter traits due to permanent environmental effects ranged from 0.02 to 0.20 (Table 5). In general, estimates of repeatabilities ranged from 0.11 to 0.20 for both LR and LW and for both regions. Estimates of repeatability for NM and FI ranged from 0.02 to 0.07. For both breeds, higher repeatabilities were observed in the CA data for TNB, NBA, and NSB compared to the AS data. For LR, estimates of repeatability for CA were 0.20 ± 0.01, 0.18 ± 0.01, and 0.20 ± 0.01 for TNB, NBA, and NSB, respectively, and 0.12 ± 0.01,0.11 ± 0.01, and 0.08 ± 0.02 for TNB, NBA, and NSB, respectively, for AS. For LW, corresponding estimates of repeatability for CA were 0.18 ± 0.01, 0.17 ± 0.01, and 0.18 ± 0.01, and 0.14 ± 0.01, 0.15 ± 0.01, and 0.06 ± 0.01 for AS.

Service sire effects were consistently low for both LR and LW and for both regions, ranging from 0% to 5% (Table 5). The larger estimates were for TNB and NBA, ranging from 2% to 5%.

## Correlations Between Traits Within Regions

**Figure 4.**
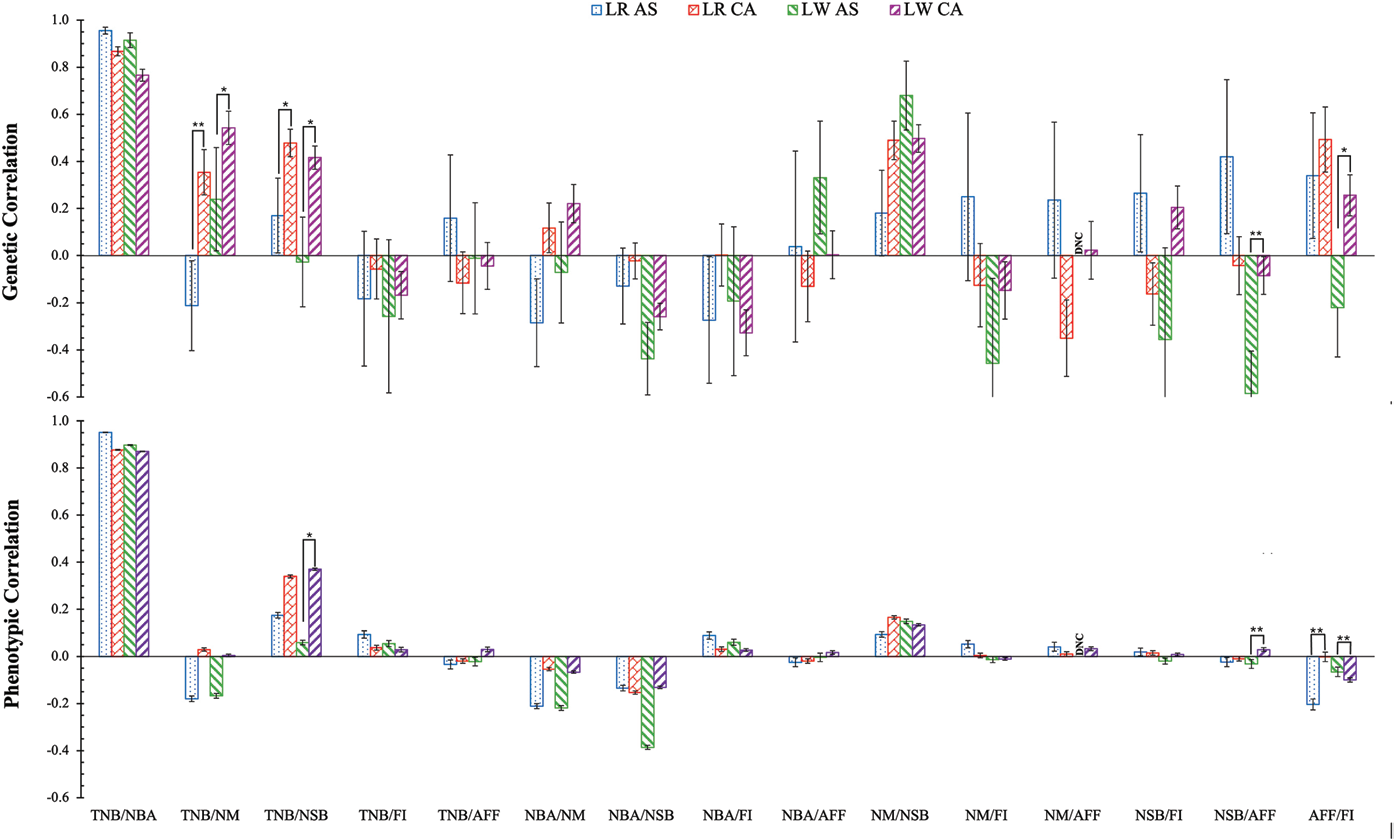
Estimates (and standard error bars) of genetic and phenotypic correlations among reproduction traits^1^ within Canada (CA) and Southeast Asia (AS) and by breed (Landrace, LR; Large White, LW) based on bivariate models. *: *P <* 0.05, **: *P <* 0.01. ^1^ Total number born (TNB), number born alive (NBA), number of mummified (NM), number of stillborn (NSB), farrowing interval (FI), age at first farrowing (AFF). DNC: did not converge.

Estimates of genetic and phenotypic correlations within CA and AS for the 6 reproduction traits are in Figure 4 and Figure S2. Estimates of the genetic correlation between TNB and NBA were very high for all regions, ranging from 0.77 to 0.96. Estimates of genetic correlations between TNB and at-birth mortality traits (NSB and NM) were lower and varied significantly between regions. In CA, the estimate of the genetic correlation between TNB and NSB was 0.48 ± 0.06 for LR and 0.42 ± 0.05 for LW, significantly (*P* < 0.05) higher than corresponding estimates in AS, which were 0.17 ± 0.16 for LR and −0.03 ± 0.19 for LW. However, within AS, these estimates were not significantly different from zero and from each other. Similarly, for TNB and NM, the CA data showed estimates of 0.35 ± 0.10 for LR and 0.54 ± 0.07 for LW, which contrasts with lower estimates (*P* < 0.05) based on the AS data (−0.21 ± 0.19 for LR and 0.24 ± 0.22 for LW). Furthermore, estimates of the genetic correlation between NSB and AFF were notably different between regions for LW (*P* < 0.01), with a nonsignificant estimate in the CA data (−0.08 ± 0.08), compared to a moderate negative estimate based on the AS data (−0.59 ± 0.18). For LR, estimates of the genetic correlation between NSB and AFF were not significantly different between the CA and AS data, with −0.04 ± 0.12 in CA and 0.42 ± 0.33 in AS. Interestingly, estimates of the genetic correlation between AFF and FI differed between breeds in the 2 regions. For LR, moderate positive genetic correlations were estimated between AFF and FI in both regions (0.49 ± 0.14 in CA and 0.34 ± 0.27 in AS). For LW, the signs of estimates of the genetic correlation between AFF and FI were opposite for the 2 regions, with an estimate of 0.26 ± 0.09 in CA and −0.22 ± 0.21 in AS.

Estimates of phenotypic correlations showed similar trends as estimates of genetic correlations. However, they were closer to zero for most pairs of traits, except for high phenotypic correlations between TNB and NBA.

### Correlations between parities within regions

Estimates of heritabilities and of genetic and phenotypic correlations of a given trait between parities by region are presented in Tables 6 and 7. For heritabilities, estimates from the multivariate model exhibited varying trends with parity numbers across traits, breeds, and regions. For the CA data, heritability estimates for NBA slightly increased with parity numbers for both breeds. The same trend was found for LW in AS herds but not for LR, which could be explained by the lower repeatability estimated for NBA for LR in the AS data with the univariate repeatability model (Table 5). For all traits, estimates of genetic correlations between parities ranged from moderate to high (0.43 to 0.99) for both regions and both breeds (Tables 6 and 7). For all traits, estimates of genetic correlations between parities in the AS data were not significantly different from 1 for either breed. For the CA data, estimates of genetic correlations between parity 2 and later parities were also not significantly different from 1 for most traits and for either breed, with an average estimate of 0.77. However, estimates of genetic correlations between parity 1 and later parities were found to be significantly different from 1 for most traits, ranging from 0.43 to 0.95 for both LR and LW. Estimates of genetic correlations between parities for NM were not significantly different from 1 for either breed or region, except between parities 2 and 3 for LR in CA. For TNB and NBA, estimates of genetic correlations between parities were higher in the CA data compared to the AS data for LR and relatively similar between the 2 regions for LW. For NM, estimates of genetic correlations between parity 2 and later parities were lower for LR than for LW for both regions. Estimates of genetic correlations for NM for LW were slightly higher in the CA than in the AS data. Estimates of genetic correlations for NSB in the AS data are not shown for LW because of model convergence problems but for LR, the same trends as described above for NM were observed for both regions. Moderate to high estimates of genetic correlations between parities were found for NSB for LW in the CA data, ranging from 0.72 to 0.93.

**Table 6.** Estimates (standard errors in parentheses) of heritabilities (bold, on diagonal) and of genetic (above diagonal) and phenotypic (below diagonal) correlations between measurements of each reproduction trait for different parities in the Canadian data for 2 breeds

Breed	Landrace								Large white							
Traits/Parity	1		2		3		4		1		2		3		4	
Total number born																
1	**0.10**	(0.02)	0.85	(0.09)	0.68^*^	(0.11)	0.87	(0.12)	**0.07**	(0.01)	0.50^*^	(0.09)	0.73^*^	(0.12)	0.77^*^	(0.08)
2	0.20	(0.01)	**0.10**	(0.02)	0.95	(0.11)	0.92	(0.14)	0.17	(0.01)	**0.10**	(0.01)	0.73^*^	(0.11)	0.92	(0.07)
3	0.17	(0.02)	0.23	(0.02)	**0.15**	(0.03)	0.88	(0.14)	0.12	(0.02)	0.18	(0.02)	**0.09**	(0.06)	0.84	(0.13)
4	0.16	(0.03)	0.18	(0.03)	0.19	(0.03)	**0.15**	(0.03)	0.16	(0.01)	0.21	(0.01)	0.25	(0.01)	**0.11**	(0.02)
Number born alive																
1	**0.07**	(0.01)	0.90	(0.11)	0.66^*^	(0.15)	0.80	(0.17)	**0.06**	(0.01)	0.43^*^	(0.11)	0.52^*^	(0.15)	0.89	(0.07)
2	0.18	(0.01)	**0.09**	(0.02)	0.85	(0.15)	0.89	(0.18)	0.16	(0.01)	**0.08**	(0.01)	0.67^*^	(0.15)	0.73^*^	(0.10)
3	0.15	(0.02)	0.19	(0.02)	**0.09**	(0.03)	0.96	(0.17)	0.11	(0.02)	0.17	(0.02)	**0.09**	(0.02)	0.81	(0.14)
4	0.12	(0.03)	0.12	(0.03)	0.16	(0.03)	**0.11**	(0.04)	0.15	(0.01)	0.19	(0.01)	0.23	(0.01)	**0.10**	(0.02)
Number mummified																
1	**0.04**	(0.01)	0.85	(0.26)	0.94	(0.28)	0.68	(0.33)	**0.03**	(0.01)	0.90	(0.22)	0.60	(0.22)	0.78	(0.20)
2	0.06	(0.01)	**0.03**	(0.04)	0.78^*^	(0.04)	0.50	(0.37)	0.05	(0.01)	**0.02**	(0.01)	0.84	(0.26)	0.91	(0.24)
3	0.02	(0.02)	0.06	(0.02)	**0.02**	(0.02)	0.99	(0.36)	0.02	(0.02)	0.01	(0.02)	**0.05**	(0.02)	0.74	(0.23)
4	0.03	(0.02)	0.06	(0.03)	0.07	(0.03)	**0.05**	(0.03)	0.06	(0.01)	0.07	(0.01)	0.05	(0.02)	**0.04**	(0.01)
Number still born																
1	**0.11**	(0.02)	0.70^*^	(0.10)	0.82	(0.12)	0.87	(0.13)	**0.11**	(0.01)	0.79^*^	(0.06)	0.72^*^	(0.08)	0.87	(0.07)
2	0.17	(0.01)	**0.06**	(0.02)	0.76	(0.16)	0.56^*^	(0.20)	0.17	(0.01)	**0.14**	(0.02)	0.77^*^	(0.08)	0.91	(0.06)
3	0.19	(0.02)	0.21	(0.02)	**0.06**	(0.02)	0.47^*^	(0.24)	0.17	(0.02)	0.20	(0.02)	**0.16**	(0.03)	0.93	(0.07)
4	0.22	(0.03)	0.15	(0.03)	0.23	(0.03)	**0.14**	(0.04)	0.18	(0.01)	0.22	(0.01)	0.21	(0.02)	**0.13**	(0.02)

^*^: significantly different from 1 at *P* < 0.05.

**Table 7.** Estimates (standard errors in parentheses) of heritabilities (bold, on diagonal) and of genetic (above diagonal) and phenotypic (below diagonal) correlations between measurements of each reproduction trait for different parities in the Southeast Asia data

Breed	Landrace						Large White					
Trait/Parity	1		2		>2		1		2		>2	
Total number born												
1	**0.09**	(0.03)	0.95	(0.22)	0.64	(0.31)	**0.05**	(0.02)	0.79	(0.26)	0.91	(0.30)
2	0.12	(0.02)	**0.05**	(0.02)	0.48	(0.34)	0.13	(0.02)	**0.06**	(0.03)	0.70	(0.30)
> 2	0.10	(0.03)	0.16	(0.03)	**0.08**	(0.08)	0.12	(0.03)	0.21	(0.02)	**0.06**	(0.03)
Number born alive												
1	**0.07**	(0.03)	0.94	(0.24)	0.83	(0.35)	**0.03**	(0.02)	0.53	(0.33)	0.93	(0.29)
2	0.10	(0.02)	**0.08**	(0.29)	0.61	(0.31)	0.13	(0.02)	**0.07**	(0.03)	0.75	(0.23)
>2	0.10	(0.03)	0.16	(0.03)	**0.07**	(0.04)	0.14	(0.03)	0.21	(0.02)	**0.08**	(0.04)
Number mummified												
1	**0.01**	(0.01)	0.64	(1.13)	0.98	(1.36)	**0.04**	(0.02)	0.68	(0.32)	0.75	(0.28)
2	0.06	(0.02)	**0.04**	(0.03)	0.51	(0.48)	0.05	(0.02)	**0.05**	(0.02)	0.67	(0.33)
>2	0.03	(0.03)	0.04	(0.03)	**0.05**	(0.03)	0.05	(0.03)	0.04	(0.03)	**0.09**	(0.04)
Number still born												
1	**0.04**	(0.02)	0.54	(0.35)	0.58	(0.62)	**0.00**	(0.00)	-	(-)	-	(-)
2	0.06	(0.03)	**0.08**	(0.03)	0.99	(0.60)	0.01	(0.02)	**0.02**	(0.02)	0.33	(0.54)
>2	0.10	(0.03)	0.00	(0.03)	**0.02**	(0.02)	-0.01	(0.03)	0.06	(0.03)	**0.06**	(0.03)

Estimates of phenotypic correlations for TNB, NBA, and NSB between parities were slightly higher in the CA data compared to the AS data for both breeds (Tables 6 and 7). Notably, the phenotypic correlations for NSB were higher in the CA data, ranging from 0.15 to 0.23 for LR and from 0.17 to 0.22 for LW, compared to 0.001 to 0.10 for LR and −0.01 to 0.06 for LW in AS. Estimates of phenotypic correlations between parities for NM were weak and close to zero for both breeds and both regions.

### Genetic correlations between regions

**Figure 5.**
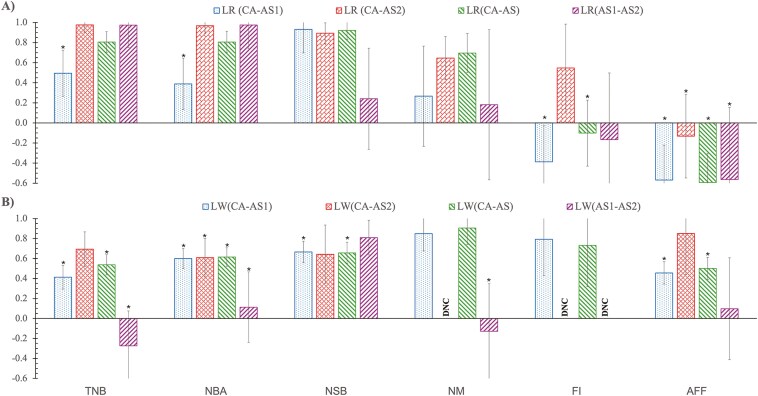
Estimates (and standard error bars) of genetic correlations for reproduction traits between Canada (CA) and each and the combined Southeast Asia herds^1^ (AS1, AS2, AS) for A) Landrace (LR) and B) Large White (LW) pigs. ^1^ Genetic correlation between a pair of regions: CA-AS1, Canadian herds and Southeast Asian herd 1; CA-AS2, Canadian herds and Southeast Asian herd 2; AS1-AS2, Southeast Asian herd 1 and Southeast Asian herd 2; CA-AS, Canadian herds and 2 Southeast Asian herds. *: significantly different from 1 at *P* < 0.05. DNC: did not converge.

Estimates of genetic correlations for reproduction traits between regions are in Figure 5 and Table S1. Estimates of genetic correlations between CA and AS differed between LR and LW for some traits. For LR, estimates of genetic correlations for TNB, NBA, and NSB, were not significantly different from 1, with values of 0.81 ± 0.10, 0.81 ± 0.10, and 0.92 ± 0.10, respectively. For LW, estimates of genetic correlations for TNB, NBA, and NSB were moderately positive and significantly different from 1 with 0.54 ± 0.10 for TNB, 0.69 ± 0.10 for NBA, and 0.66 ± 0.11 for NSB. However, the estimate of the genetic correlation between CA and AS for NM was slightly higher for LW than for LR (0.90 ± 0.17 and 0.70 ± 0.20, respectively) and both were not significantly different from 1. The estimate of the genetic correlation for FI of LR between CA and AS was negative and significantly different from 1 (−0.10 ± 0.33) but positive and not significantly different from 1 for LW (0.73 ± 0.41). Similarly, for AFF, estimates of the genetic correlation between CA and AS were −0.59 ± 0.29 for LR and 0.50 ± 0.11 for LW and these estimates were significantly different from 1. In a further analysis of genetic correlations between CA and AS for AFF, using the AS dataset that included only non-imported gilts (Figure S3), the signs of genetic correlations remained opposite between LR and LW. For LR, the estimate of genetic correlation was negative and significantly different from 1 (−0.96 ± 0.34), whereas for LW, it was highly positive and not significantly different from 1 (0.94 ± 0.38).

Estimates of genetic correlations between CA and each AS herd (AS1, AS2) showed a divergence in results between the 2 AS herds (Figure 5 and Table S1). For LR, across traits, estimates of the genetic correlation between CA and AS2 were highly positive and not significantly different from 1, except for AFF, but corresponding estimates between CA and AS1 were significantly different from 1 across traits, except for NSB and NM. For LW, the same trend was observed for estimates of genetic correlations between CA and AS2, with most traits showing no significant differences from 1, except for NM and FI, while estimates of the genetic correlation between CA and AS1 were significantly different from 1 for TNB, NBA, NSB, and AFF. For NSB, estimates of genetic correlations were found to be slightly higher between CA and AS1 than between CA and AS2 for both breeds. For NM, the estimate of the genetic correlation between CA and AS1 was higher for LW than for LR. For FI, the estimate of the genetic correlation between CA and AS1 had a different sign for the 2 breeds, with it being negative and significantly different from 1 for LR (−0.39 ± 0.36), but positive and not significantly different from 1 for LW (0.79 ± 0.36). Estimates of the genetic correlation between CA and AS2 for FI are not shown for LW due to convergence problems, but a moderate positive estimate, though not significantly different from 1, was found for LR (0.55 ± 0.44). For AFF, the estimate of the genetic correlation between CA and AS1 differed in sign between the 2 breeds (-0.57 ± 0.35 for LR and 0.46 ± 0.11 for LW) and estimates were significantly different from 1 for both breeds. The estimate of the genetic correlation between CA and AS2 for AFF was highly positive and not significantly different from 1 for LW (0.85 ± 0.41), but negative and significantly different from 1 for LR (−0.13 ± 0.42). In further analyses of genetic correlations between CA and AS1, using only non-imported gilts in the AS1 dataset (Figure S3), the signs of genetic correlations for AFF and FI remained opposite between LR and LW. For the estimates between CA and AS2, negative genetic correlations were found for both AFF and FI in LR. Estimates for FI did not converge.

For all traits, estimates of genetic correlations between the 2 AS herd, AS1 and AS2, were generally higher for LR than for LW, except for NSB and AFF (Figure 5 and Table S1). For LR, estimates of genetic correlations between the 2 AS herds were not significantly different from 1 for all traits, except for AFF, while estimates were significantly different from 1 for all traits for LW, except for NSB and AFF. The sign of estimates of the genetic correlation between AS1 and AS2 for AFF also differed between the 2 breeds, with a negative estimate for LR (−0.56 ± 0.72) and a positive estimate for LW (0.1 ± 0.51). These estimates were, however, not significantly different from each other due to high SE.

## Discussion

In this study, we estimated variance components and genetic correlations between reproduction traits recorded on sows from Western breeds in temperate (Canada) and tropical (Southeast Asia) climates. We were particularly interested in estimates of genetic correlations of reproduction traits between Canada and Southeast Asia herds, as these allow us to quantify GxE interactions between 2 climatic environments (temperate versus tropical). The results indicated some clear differences in reproductive performance and variance components between the Canadian and Southeast Asian data, and in genetic correlations between reproduction traits within each region. Marked reductions in litter performance traits were observed under the Southeast Asian climate, which is characterized by higher average temperatures and humidity throughout the year. Several studies have described the negative influence of high temperatures on the reproductive performance of sows (Suriyasomboon et al., 2006; Sevillano et al., 2016).

Estimates of variance components and heritabilities of reproductive traits were also found to differ between Canada and Southeast Asia. Genetic correlations for each reproduction trait between Canada and Southeast Asia were significantly different from 1 for most traits for both breeds, indicating the presence of GxE interactions between these 2 regions. Although there was no evidence of differences in the drop of reproductive performance from temperate to tropical climates, nor were there differences in seasonal trends in contemporary group effect estimates within Canada and Southeast Asia between the LR and LW breeds, the higher estimates of genetic correlations between regions for the LR breeds suggest it to have greater robustness and adaptability to tropical climates. This agrees with previous studies of differences in performance between these 2 breeds. Under the tropical climate in Thailand, Tantasuparuk et al. (2000) found that Landrace has an overall higher reproductive performance compared to LW. This indicates that the LR breed may be a more suitable choice for breeding programs in tropical countries, aiming to optimize reproductive performance under varying climatic conditions.

### Genetic parameters

In this study, heritability estimates of the 6 reproductive traits ranged from 0.02 to 0.14 across breeds and regions. Heritability estimates for TNB, NBA, and NSB were slightly higher in the Canadian data for both breeds, ranging from 0.07 to 0.13, compared to estimates that ranged from 0.05 to 0.07 for the Southeast Asian data. Estimates of heritability for TNB, NBA, and NSB align well with earlier estimates for the same breeds using a repeatability model in a humid tropical climate, which ranged from 0.05 to 0.16 (Ogawa et al., 2019; Zhang et al., 2020; Yang et al., 2023) and in temperate climates, which ranged from 0.06 to 0.18 (Southwood and Kennedy, 1990; Szostak and Katsarov, 2013). Estimates of repeatability for TNB and NBA were relatively moderate, ranging from 0.15 to 0.20, and again, were found to be higher in the Canadian data. Previous studies also reported repeatabilities for TNB and NBA to range from 0.09 to 0.20 (Hanenberg et al., 2001; Zhang et al., 2020). Moderately high repeatabilities were estimated for NSB in the Canadian data, 0.20 for LR and 0.18 for LW, compared to low repeatabilities in the Southeast Asia data, 0.08 for LR and 0.06 for LW. Previous studies have reported repeatability estimates for NSB in the range from 0.02 to 0.14 (Hanenberg et al., 2001; Imboonta et al., 2007; Ogawa et al., 2019; Yang et al., 2023). The higher repeatability estimates for TNB, NBA, and NSB in the Canadian versus the Southeast Asian data indicate the smaller effect of temporary environmental factors on these traits in the Canadian data. This could be the result of differences in management, climate, and trait measurements between these regions (see, Material and Methods). For NM, estimates of heritability were low (0.03) and consistent for the 2 regions, which agrees with estimates of previous studies (Corredor et al., 2020; Zhang et al., 2020). Heritability estimates for FI were 0.07 for LR for both regions, and 0.02 in Southeast Asia and 0.05 in Canada for LW, which are all in the range of previous studies, from 0.04 to 0.16 (Serenius et al., 2004; Cavalcante Neto et al., 2009; Tomiyama et al., 2011). For LR, heritability estimates for AFF were consistent between regions, but they were substantially higher in Southeast Asia (0.40) than in Canada (0.14) for LW. The high estimate for AFF for LW in the Southeast Asia data was caused by the data from AS1, which showed an estimate of heritability of 0.45 ± 0.05, compared to 0.06 ± 0.05 for AS2. Previous studies also reported a wide range of estimated heritability for this trait, ranging from low (0.16; Serenius et al., 2004; Zhang et al., 2020) to moderate (0.23; Knauer et al., 2011) and high (0.44;  Cavalcante-Neto et al., 2011). However, in this study, the much higher estimates of heritability for AFF for LW gilts in AS1 compared to AS2 were primarily driven by apparent different management of imported LW gilts in AS1. Notably, 57.9% of LW gilts in AS1 were imported, compared to 25.3% of LW gilts in AS2, 12.6% of LR gilts in AS1, and 31.1% of LR gilts in AS2 (Table 2). The imported LW gilts in AS1 exhibited a significantly higher estimate of heritability for AFF (0.84 ± 0.08; Table S3) compared to non-imported gilts (0.19 ± 0.07; Table S3). This marked difference in heritability estimates is likely attributable to specialized management practices for the imported gilts in AS1, such as synchronization or extended quarantine procedures for imported animals, noting the larger average AFF for imported gilts (350 vs. 329 d for non-imported gilts). Such practices can minimize environmental variance, as evidenced by the significantly lower residual variance estimated in AS1 for imported (39.1 ± 21.6 d) compared to non-imported LW gilts (147.7 ± 13.3 d). Additionally, the additive genetic variance of AFF was notably higher for imported LW gilts in AS1 (226.2 ± 32.0 d) than for non-imported gilts (37.3 ± 14.0 d), supporting the idea that these management strategies possibly mitigated environmental impacts and increased genetic variance. The relatively low estimate of the genetic correlation between imported and non-imported gilts for AFF (0.36 ± 0.21) for LW in AS1 further underscores the distinct genetic influences affecting AFF for these 2 groups of gilts.

In general, the observed differences in estimates of heritability and repeatability between the Canadian and Southeast Asian data can be due to the differences in environmental factors (i.e., climate and management) and differences in the method and accuracy of measurement. These differences in estimates of heritability for reproduction traits indicate the possibility of GxE for reproductive performance between the Canadian and Southeast Asian herd environments.

Proportions of phenotypic variance explained by service sire were consistently low across traits, breeds, and regions, ranging from 0.00 to 0.05. A small contribution of service sire has also been reported in the literature, with estimates ranging from 0.00 to 0.04 (Chen et al., 2003; Su et al., 2007; Wolf and Wolfova, 2012).

### Genetic correlations between traits within regions

Estimates of the genetic correlation between TNB and NBA were close to one for both regions, ranging from 0.77 to 0.96 (Figure 4, Figure S2). This agrees with previous studies, which reported high genetic correlations (>0.7) between these 2 traits (Roehe and Kennedy, 1995; Imboonta et al., 2007; Zhang et al., 2020). Low to moderate positive estimates were found for the genetic correlation between TNB and NSB in both regions, ranging from −0.03 to 0.48 for both LR and LW. Previous studies have reported genetic correlations between these 2 traits that ranged from 0.23 to 0.40 (Hanenberg et al., 2001; Serenius et al., 2004; Imboonta et al., 2007; Ogawa et al., 2019). Low negative to moderate positive genetic correlation estimates were also found between TNB and NM in both regions, ranging from 0.24 to 0.54 for both LR and LW, except for LR in Southeast Asian herds, which showed a negative genetic correlation estimate (−0.21 ± 0.19). Ye et al. (2018) estimated a low genetic correlation between TNB and NM of 0.13 for LW under a subtropical climate in China. Under tropical climates in the Southwest of China, Yang et al. (2023) also reported low to moderately high genetic correlation estimates between TNB and NM of 0.21 for LR and 0.49 for LW. This indicates a more unfavorable relationship between TNB and NM under tropical climates.

Estimates of genetic correlations of FI with other reproduction traits in both regions, as well as of AFF with other traits in both regions, had high SE, indicating substantial uncertainty in these estimates. For Canadian data, favorable low to moderate negative genetic correlations between FI and litter size traits were found, consistent with results from previous studies (Tomiyama et al., 2011; Zhang et al., 2020). This indicates that selection to increase TNB and NBA can potentially reduce FI. In addition, low to moderate negative estimates of genetic correlations between FI and perinatal mortality traits (NM and NSB) were found for both breeds in the Canadian data, except between FI and NSB for LW, with a positive estimate of 0.20 ± 0.09. This indicates a generally unfavorable genetic relationship between FI and NSB for LW in Canada, as an increase in FI could lead to an increase in NSB. Zhang et al. (2020) estimated the genetic correlation between FI and NSB to be −0.18 for LR and −0.01 for LW from farms across China. It should be noted that NSB is difficult to accurately evaluate in a barn setting without 24-h supervision of farrowing. In such settings, many litters are not observed until 6 to 12 h after the start of farrowing and all pigs found dead when the litter is first observed are counted as stillborn. Accurate and consistent evaluation of this trait will also differ between settings.

Estimates of genetic correlations between AFF and litter size traits (TNB and NBA) were found to be low negative to low positive in both regions, which agrees with previous reports of these correlations, which ranged from −0.01 to −0.22 (Ye et al., 2018; Zhang et al., 2020). This indicates that selection to increase TNB and NBA potentially decreases AFF. Estimates of genetic correlations between AFF and NSB were negative for both breeds and regions, except for LR in Southeast Asia, which was positive but with a high standard error. Zhang et al. (2020) estimated the genetic correlation between AFF and NSB in China to be 0.03 for LR and 0.41 for LW. Estimates of genetic correlations between FI and NM and between AFF and FI were found to be low negative in both regions for LW. For LR, estimates of genetic correlations of NM with AFF and FI were found to have different signs between the 2 regions; both were negative in Canada but positive in Southeast Asia. To the best of our knowledge, no previously reported estimates of genetic correlations of FI with NM and of AFF with NM are available. Estimates of genetic correlations between AFF and FI were moderately positive for both breeds and regions, except for LW in Southeast Asia (−0.22 ± 0.21). Zhang et al. (2020) reported moderate positive correlations between these 2 traits, 0.17 for LR and 0.16 for LW. Our study showed a stronger genetic correlation between these traits for LR in both regions and for LW in Canada, ranging from 0.26 to 0.49, which indicates a favorable relationship, such that selecting for lower FI is expected to decrease AFF and versa.

Estimates of genetic correlations for reproductive traits between parities within each region ranged from moderate to high positive (0.43 to 0.99). Notably, for Asian herds, estimates of genetic correlation between parities were not significantly different from 1 for all traits. For Canada, estimates of genetic correlations between parities were mostly consistent and not significantly different from 1 between parity 2 and later parities but they were significantly different from 1 between parity 1 and later parities. In the literature, genetic correlations between parities always exceed 0.7, except in some cases between parity 1 and later parities (Roehe and Kennedy, 1995; Hanenberg et al., 2001; Ye et al., 2018). In this study, we found the genetic correlation between parity 1 and later parities to be significantly different from 1 in the Canadian data for most traits for both LR and LW. This indicates that reproductive traits in sows should be analyzed using a multivariate model that considers parity 1 as a separate trait, rather than using a simple repeatability model, which assumes 1) homogeneity of variance across parities, 2) genetic correlations between parities equal to 1, and 3) equal residual variances and co-variances between parities (Roehe & Kennedy, 1995).

### Genetic correlations between regions

Estimates of the genetic correlation of the same trait recorded under different climatic environments can be used to determine whether GxE is present (Falconer, 1952). In this context, our study revealed some significant GxE for both breeds and for most reproduction traits, with estimates of the genetic correlation between temperate (Canada) and tropical (Southeast Asia) climatic environments that were significantly different from 1.

Estimates of genetic correlations for CA with AS1, AS2, and the combined AS data for litter size traits were moderate to high (Figure 5). The higher estimates for LR compared to LW indicate that LR sows tend to adapt better to tropical climate conditions. For NSB, estimates of the genetic correlation for CA with AS and the 2 individual AS herds were not significantly different from 1 for LR. For LW, estimates of the genetic correlation for NSB between CA and AS were moderate and significantly different from 1 for CA with AS and AS1. As expected, the genetic correlation between CA and AS was also estimated to be high for TNB for both LR and LW, as TNB is calculated as the sum of NBA and NSB. Estimates of genetic correlations for NM between regions varied from low to high for both breeds but with notably high SE. Interestingly, genetic correlation estimates between CA and AS for AFF and FI had opposite signs for the LR and LW breeds. The LR breed exhibited negative genetic correlation estimates for these traits between CA and AS, while moderate to high positive genetic correlation estimates were found for LW. This indicates that the LR and LW breeds may respond differently to environmental factors that affect these traits.

For all traits, estimates of the genetic correlation between AS1 and AS2 were found to be high and not significantly different from 1 for LR (except for AFF), which indicates no clear evidence for GxE between 2 Southeast Asian herds for this breed, although SE were large. However, for LW, estimates of the genetic correlation between AS1 and AS2 were significantly different from 1 for TNB, NBA, and NM, which indicated the presence of GxE between the 2 Southeast Asian herds. The presence of GxE between the 2 Southeast Asian herds may be explained by differences in environmental factors other than climate, including housing, feed, health status, and management, as well as the accuracy of phenotype measurements. Generally, estimates of genetic correlations between AS1 and AS2 were associated with higher SE than estimates between CA and AS because of a smaller number of records. Additionally, estimates of genetic correlations between CA and AS2 for litter size traits (TNB and NBA) were higher and not significantly different from 1 (except for NBA for LW) for both LR and LW, compared to corresponding estimates between CA and AS1. Thus, the level of GxE for reproduction traits was lower for CA versus AS2 compared to CA versus AS1.

The presence of GxE for reproduction traits between Canada and Southeast Asia indicates that there may be benefits to incorporate data from tropical environments into the genetic evaluation of Western purebreds to improve overall reproduction performance and adaptability when exposed to tropical climate conditions.

To assess whether seasonal THI differences within Canada (Figure 1) could help predict reproductive performance in Southeast Asian herds, we conducted further analyses of the Canadian data by estimating genetic correlations for reproduction traits between the warm (from June to September) and cold (from October to May) seasons within Canada and with data from the Southeast Asian herds (Figures S5–S7). Within Canada, estimates of genetic correlations between warm and cold seasons were high and not significantly different from 1 (Figure S5) for TNB, NBA, NSB, NM, and FI for both breeds. Estimates of genetic correlations of performance from the warm season in CA with that in AS herds were slightly higher than estimates of genetic correlations between CA-cold season and AS herds for TNB, NBA, NSB, and NM. However, these differences were not statistically significant. Additionally, these results were consistent with our main findings for the presence of GxE interaction between CA and AS for reproductive traits, indicating that the season in Canada did not significantly affect the genetic correlations for these traits with Southeast Asia in our current datasets. Moreover, in all further analyses, estimates of genetic correlations for AFF between different environmental conditions (Figures S5-S7) were found to be significantly different from 1, ranging from low to moderate for both LR and LW. Overall, these analyses suggest that even under similar temperature conditions (e.g., warm season in Canada and tropical climate in Southeast Asia), there is still a presence of GxE interaction between the 2 regions. This implies that factors beyond average daily THI, including diurnal THI variation, specific on-site management practices, and local feedstuff differences between regions, likely contribute significantly to the observed GxE interactions. These findings highlight the complexity of the GxE interactions between regions, underscoring the importance of considering multiple environmental parameters when developing cross-regional breeding strategies. Future research should quantify the relative contributions of these factors to optimize breeding programs for consistent performance across diverse climatic conditions.

The current study included only purebred data. However, it is important to note that the main goal of most breeding programs is to improve the performance of crossbreds in commercial herds. Even under the same climatic environment, previous studies have reported estimates of genetic correlations between purebred and crossbred to be lower than 1 for both production and reproduction traits (Wientjes and Calus, 2017; Godinho et al., 2019; Esfandyari et al., 2020; Kramer et al., 2021). Thus, in addition to genotype-by-genotype interactions between purebreds and crossbreds, it is also critical to investigate and quantify the level of GxE between purebreds in temperate climates and crossbreds in tropical climates, and vice versa.

## Conclusions

In this study, estimates of genetic correlations for most reproductive traits of purebred LR and LW sows between temperate (CA) and tropical (AS) climates were found to be significantly different from 1, indicating the presence of GxE for these Western LR and LW breeds. Differences in these estimates can be due to the differences in multiple environmental factors between regions (i.e., health status, farm management, and climate conditions between regions). Our estimates of genetic correlations between regions indicate that selecting animals based on records from tropical climates or incorporating such data into genetic evaluation models for temperate-climate animals could significantly enhance their adaptability and robustness when exposed to tropical conditions. In addition, although there was no difference in drop in performance between Canada and Southeast Asia between the 2 breeds, nor was a seasonal effect on performance within Canada more pronounced for LW than for LR, the higher genetic correlation estimates for reproduction traits between Canada and Southeast Asia that were identified for LR compared to LW indicates that LR sows may be more robust when exposed to a tropical climate.

## Supplementary Material

skaf191_suppl_Supplementary_Figures_S1-S7_Tables_S1-S3

## Abbreviations

AFF: age at first farrowing
AS: South-East Asia
CA: Canada
FI: farrowing interval
GxE: genotype-by-environment interactions
LR: Landrace
LW: Large White
NBA: number born alive
NM: number mummified
NSB: number stillborn
TNB: total number born
