# Supplementary material for: Genotype by environment interactions for reproductive performance of North American purebred sows between North America and Southeast Asia
Source: J Anim Sci. 2025 Jun 19;103:skaf191. doi: 10.1093/jas/skaf191 (PMC12272058; doi:10.1093/jas/skaf191)
Supplement: skaf191_suppl_Supplementary_Figures_S1-S7_Tables_S1-S3 [file skaf191_suppl_supplementary_figures_s1-s7_tables_s1-s3.zip › Supplementary material/Supplementary material.docx]

**List of figures (titles and captions)**

Figure S1: Daily average and range^1^ of THI in two Southeast Asian herds (AS1 and AS2) and in Canada (CA) from 2015 (A) to 2023 (I).

Captions:

^1^Daily THI range was calculated as the difference between the daily maximum THI and minimum THI values.

^*^Max: Maximum THI; Min: Minimum THI.

Figure S2: Heatmap of estimates (standard errors in parentheses) of genetic (above diagonal) and phenotypic (below diagonal) correlations among analyzed traits: total number born (TNB), number born alive (NBA), number mummified (NM), number stillborn (NSB), farrowing interval (FI), age at first farrowing (AFF) for Southeast Asian herds Landrace (A), Large White (C); for Canadian herds Landrace (B), Large White (D).

Figure S3: Estimates (and standard error bars) of genetic correlations for reproduction traits between Canada (CA) and each and the combined Southeast Asia herds^1^ (AS1, AS2, AS) for A) Landrace (LR) and B) Large White (LW) non-imported pigs.

Captions:

^1^ Genetic correlation between a pair of regions: CA-AS1, Canadian herds and Southeast Asian herd 1; CA-AS2, Canadian herds and Asian herd 2; AS1-AS2, Southeast Asian herd 1 and Southeast Asian herd 2; CA-AS, Canadian herds and two Southeast Asian herds.

*: significantly different from 1 at P < 0.05

DNC: did not converge.

Figure S4: Estimates (and standard error bars) of genetic correlations for reproduction traits between imported and non-imported gilts in each and the combined Southeast Asian herds (AS1, AS2, AS) for A) Landrace (LR) and B) Large White (LW) pigs.

Captions:

*: significantly different from 1 at P < 0.05

DNC: did not converge.

Figure S5: Estimates (with standard error bars) of genetic correlations for reproductive traits between animals phenotyped during the hot and cold seasons in Canada for Landrace (LR) and Large White (LW) pigs.

Captions:

*: significantly different from 1 at P < 0.05

DNC: did not converge.

Figure S6: Estimates (with standard error bars) of genetic correlations^1^ for reproductive traits between animals phenotyped during the hot season in Canada (CA) and each and combined Southeast Asian herds (AS1, AS2, AS) for both Landrace (LR) and Large White LW) pigs.

Captions:

^1^Genetic correlation between a pair of regions: CA-AS1, Canadian herds and Southeast Asian herd 1; CA-AS2, Canadian herds and Asian herd 2; AS1-AS2, Southeast Asian herd 1 and Southeast Asian herd 2; CA-AS, Canadian herds and two Southeast Asian herds

*: significantly different from 1 at P < 0.05

DNC: did not converge.

Figure S7: Estimates (with standard error bars) of genetic correlations^1^ for reproductive traits between animals phenotyped during the cold season in Canada (CA) and each and combined Southeast Asian herds (AS1, AS2, AS) for both Landrace (LR) and Large White LW) pigs.

Captions:

^1^Genetic correlation between a pair of regions: CA-AS1, Canadian herds and Southeast Asian herd 1; CA-AS2, Canadian herds and Asian herd 2; AS1-AS2, Southeast Asian herd 1 and Southeast Asian herd 2; CA-AS, Canadian herds and two Southeast Asian herds

*: significantly different from 1 at P < 0.05

DNC: did not converge.

Table S1: Estimates (standard errors in parentheses) of genetic correlations for reproductive traits in different regions^1^ (environments) for Landrace and Large White pigs.

| Breed |  | Landrace | | | |  | | | Large White | | | |
| --- | --- | --- | --- | --- | --- | --- | --- | --- | --- | --- | --- | --- |
| Trait and region^1^ |  | CA-AS1 | CA-AS2 | AS1-AS2 | CA-AS | |  | CA-AS1 | | CA-AS2 | AS1-AS2 | CA-AS |
| TNB |  | 0.49* | 0.98 | 0.97 | 0.81 | |  | 0.41* | | 0.69 | -0.27* | 0.54* |
|  |  | (0.23) | (0.08) | (0.22) | (0.10) | |  | (0.12) | | (0.17) | (0.35) | (0.10) |
| NBA |  | 0.39* | 0.97 | 0.97 | 0.81 | |  | 0.60* | | 0.61* | 0.11* | 0.61* |
|  |  | (0.26) | (0.08) | (0.24) | (0.11) | |  | (0.10) | | (0.19) | (0.35) | (0.09) |
| NSB |  | 0.93 | 0.89 | 0.24 | 0.92 | |  | 0.67* | | 0.64 | 0.81 | 0.66* |
|  |  | (0.23) | (0.10) | (0.50) | (0.09) | |  | (0.11) | | (0.29) | (0.17) | (0.11) |
| NM |  | 0.27 | 0.65 | 0.18 | 0.70 | |  | 0.85 | | **DNC** | -0.13* | 0.90 |
|  |  | (0.50) | (0.22) | (0.75) | (0.20) | |  | (0.17) | |  | (0.48) | (0.17) |
| FI |  | -0.39* | 0.55 | -0.17 | -0.10* | |  | 0.79 | | **DNC** | **DNC** | 0.73 |
|  |  | (0.36) | (0.44) | (0.66) | (0.33) | |  | (0.36) | |  |  | (0.41) |
| AFF |  | -0.57* | -0.13* | -0.56* | -0.59* | |  | 0.46* | | 0.85 | 0.10 | 0.50* |
|  |  | (0.35) | (0.42) | (0.72) | (0.29) | |  | (0.11) | | (0.41) | (0.51) | (0.11) |

*: significantly different from 1 at P < 0.05.

DNC: did not converge.

Table S2: Estimates of heritability (h^2^), repeatability ($\mathrm{rpt}$), and the proportion of phenotypic variance due to service sire (ss) for reproductive traits of Landrace imported and non-imported pigs in Southeast Asia, with standard errors in parentheses.

| Imported/non-imported | | Imported | | | | | |  | Non-imported | | | | | |
| --- | --- | --- | --- | --- | --- | --- | --- | --- | --- | --- | --- | --- | --- | --- |
| Trait^1^/Region^2^ | | AS | | AS1 | | AS2 | |  | AS | | AS1 | | AS2 | |
| TNB |  |  |  |  |  |  |  |  |  |  |  |  |  |  |
|  | $h^{2}$ | 0.10 | (0.04) | 0.03 | (0.05) | 0.10 | (0.05) |  | 0.04 | (0.02) | 0.04 | (0.02) | 0.04 | (0.03) |
|  | $\mathrm{rpt}$ | 0.13 | (0.02) | 0.10 | (0.04) | 0.14 | (0.03) |  | 0.12 | (0.02) | 0.12 | (0.02) | 0.11 | (0.04) |
|  | $\mathrm{ss}$ | 0.01 | (0.01) | 0.01 | (0.02) | 0.02 | (0.03) |  | 0.04 | (0.01) | 0.01 | (0.01) | 0.04 | (0.02) |
| NBA |  |  |  |  |  |  |  |  |  |  |  |  |  |  |
|  | $h^{2}$ | 0.08 | (0.04) | 0.02 | (0.05) | 0.07 | (0.04) |  | 0.04 | (0.02) | 0.04 | (0.02) | 0.05 | (0.03) |
|  | $\mathrm{rpt}$ | 0.11 | (0.02) | 0.09 | (0.04) | 0.11 | (0.03) |  | 0.12 | (0.02) | 0.12 | (0.02) | 0.12 | (0.04) |
|  | $\mathrm{ss}$ | 0.02 | (0.01) | 0.01 | (0.02) | 0.02 | (0.03) |  | 0.04 | (0.01) | 0.02 | (0.02) | 0.04 | (0.02) |
| NM |  |  |  |  |  |  |  |  |  |  |  |  |  |  |
|  | $h^{2}$ | 0.04 | (0.02) | 0.01 | (0.02) | 0.04 | (0.03) |  | 0.02 | (0.01) | 0.01 | (0.01) | 0.06 | (0.03) |
|  | $\mathrm{rpt}$ | 0.06 | (0.02) | - | - | 0.07 | (0.03) |  | - | - | - | - | - | - |
|  | $\mathrm{ss}$ | 0.01 | (0.01) | 0.03 | (0.03) | 0.01 | (0.04) |  | 0.01 | (0.01) | 0.01 | (0.01) | 0.00 | (0.01) |
| NSB |  |  |  |  |  |  |  |  |  |  |  |  |  |  |
|  | $h^{2}$ | 0.07 | (0.03) | 0.00 | (0.04) | 0.09 | (0.04) |  | 0.05 | (0.02) | 0.02 | (0.01) | 0.07 | (0.03) |
|  | $\mathrm{rpt}$ | 0.10 | (0.02) | 0.02 | (0.03) | 0.13 | (0.03) |  | 0.05 | (0.02) | 0.03 | (0.02) | - | - |
|  | $\mathrm{ss}$ | 0.01 | (0.01) | - | - | 0.01 | (0.04) |  | 0.00 | (0.00) | 0.00 | (0.01) | 0.00 | (0.01) |
| FI |  |  |  |  |  |  |  |  |  |  |  |  |  |  |
|  | $h^{2}$ | 0.03 | (0.03) | 0.07 | (0.04) | 0.03 | (0.03) |  | 0.04 | (0.03) | 0.06 | (0.03) | 0.07 | (0.06) |
|  | $\mathrm{rpt}$ | 0.03 | (0.03) | - |  | - | - |  | 0.13 | (0.03) | 0.14 | (0.04) | 0.23 | (0.08) |
|  | $\mathrm{ss}$ | 0.01 | (0.01) | 0.04 | (0.03) | 0.01 | (0.01) |  | 0.04 | (0.02) | 0.03 | (0.02) | 0.01 | (0.02) |
| AFF |  |  |  |  |  |  |  |  |  |  |  |  |  |  |
|  | $h^{2}$ | - | - | - | - | - | - |  | 0.07 | (0.03) | 0.07 | (0.03) | 0.08 | (0.06) |
|  | $\mathrm{rpt}$ | - | - | - | - | - | - |  | - | - | - | - | - | - |
|  | $\mathrm{ss}$ | - | - | - | - | - | - |  | - | - | - | - | 0.01 | (0.01) |

^1^ Traits: TNB = total Number Born, NBA = number born alive, NSB = number of stillborn, NM = number of mummified, FI = farrowing interval, AFF = age at first farrowing.

^2^ AS = Southeast Asia, AS1 = Southeast Asian herd 1, AS2 = Southeast Asian herd 2.

Table S3: Estimates of heritability (h^2^), repeatability ($\mathrm{rpt}$), and the proportion of phenotypic variance due to service sire (ss) for reproductive traits of Large White imported and non-imported pigs in Southeast Asia, with standard errors in parentheses.

| Imported/non-imported | | Imported | | | | | |  | Non-imported | | | | | |
| --- | --- | --- | --- | --- | --- | --- | --- | --- | --- | --- | --- | --- | --- | --- |
| Trait^1^/Region^2^ | | AS | | AS1 | | AS2 | |  | AS | | AS1 | | AS2 | |
| TNB |  |  |  |  |  |  |  |  |  |  |  |  |  |  |
|  | $h^{2}$ | 0.04 | (0.02) | 0.06 | (0.03) | 0.06 | (0.05) |  | 0.03 | 0.02 | 0.04 | (0.03) | 0.03 | (0.03) |
|  | $\mathrm{rpt}$ | 0.14 | (0.02) | 0.13 | (0.02) | 0.17 | (0.04) |  | 0.14 | 0.02 | 0.15 | (0.03) | 0.15 | (0.05) |
|  | $\mathrm{ss}$ | 0.01 | (0.01) | 0.02 | (0.02) | 0.01 | (0.02) |  | 0.06 | 0.02 | 0.01 | (0.02) | 0.08 | (0.03) |
| NBA |  |  |  |  |  |  |  |  |  |  |  |  |  |  |
|  | $h^{2}$ | 0.05 | (0.02) | 0.06 | (0.03) | 0.04 | (0.05) |  | 0.04 | 0.02 | 0.03 | (0.03) | 0.03 | (0.03) |
|  | $\mathrm{rpt}$ | 0.16 | (0.02) | 0.16 | (0.02) | 0.19 | (0.04) |  | 0.14 | 0.02 | 0.15 | (0.03) | 0.13 | (0.05) |
|  | $\mathrm{ss}$ | 0.01 | (0.01) | 0.01 | (0.02) | 0.02 | (0.02) |  | 0.05 | 0.02 | 0.02 | (0.02) | 0.08 | (0.03) |
| NM |  |  |  |  |  |  |  |  |  |  |  |  |  |  |
|  | $h^{2}$ | 0.02 | (0.01) | 0.01 | (0.02) | 0.01 | (0.03) |  | 0.02 | 0.01 | 0.03 | (0.02) | - | - |
|  | $\mathrm{rpt}$ | 0.03 | (0.02) | 0.03 | (0.01) | - | - |  | - | - | - | - | - | - |
|  | $\mathrm{ss}$ |  |  | - | - | - | - |  | - | - | - | - | - | - |
| NSB |  |  |  |  |  |  |  |  |  |  |  |  |  |  |
|  | $h^{2}$ | 0.04 | (0.02) | 0.04 | (0.02) | 0.01 | (0.03) |  | 0.03 | 0.02 | 0.04 | (0.03) | 0.01 | (0.03) |
|  | $\mathrm{rpt}$ | 0.07 | (0.02) | 0.07 | (0.02) | 0.06 | (0.04) |  | 0.12 | 0.03 | 0.17 | (0.04) | 0.11 | (0.05) |
|  | $\mathrm{ss}$ | - | - | - | - | - | - |  | 0.03 | 0.01 | 0.00 | (0.02) | 0.04 | (0.02) |
| FI |  |  |  |  |  |  |  |  |  |  |  |  |  |  |
|  | $h^{2}$ | 0.00 | (0.01) | 0.00 | (0.02) | 0.04 | (0.05) |  | 0.02 | 0.03 | 0.03 | (0.04) | - | - |
|  | $\mathrm{rpt}$ | 0.02 | (0.02) | 0.01 | (0.02) | - | - |  | 0.04 | 0.03 | 0.08 | (0.05) | - | - |
|  | $\mathrm{ss}$ | - | - | - | - | - | - |  | 0.02 | 0.02 | 0.03 | (0.04) | - | - |
| AFF |  |  |  |  |  |  |  |  |  |  |  |  |  |  |
|  | $h^{2}$ | 0.81 | (0.08) | 0.84 | (0.08) | - | - |  | 0.07 | 0.04 | 0.17 | (0.06) | - |  |
|  | $\mathrm{rpt}$ | - | - | - | - | - | - |  | - | - | - | - | - | - |
|  | $\mathrm{ss}$ | 0.07 | (0.04) | 0.00 | (0.03) | - | - |  | - | - | 0.06 | (0.04) | - | - |

^1^ Traits: TNB = total Number Born, NBA = number born alive, NSB = number of stillborn, NM = number of mummified, FI = farrowing interval, AFF = age at first farrowing.

^2^ AS = Southeast Asia, AS1 = Southeast Asian herd 1, AS2 = Southeast Asian herd 2.

Figure S1: Daily average and range^1^ of THI in two Southeast Asian herds (AS1 and AS2) and in Canada (CA) from 2015 (A) to 2023 (I).


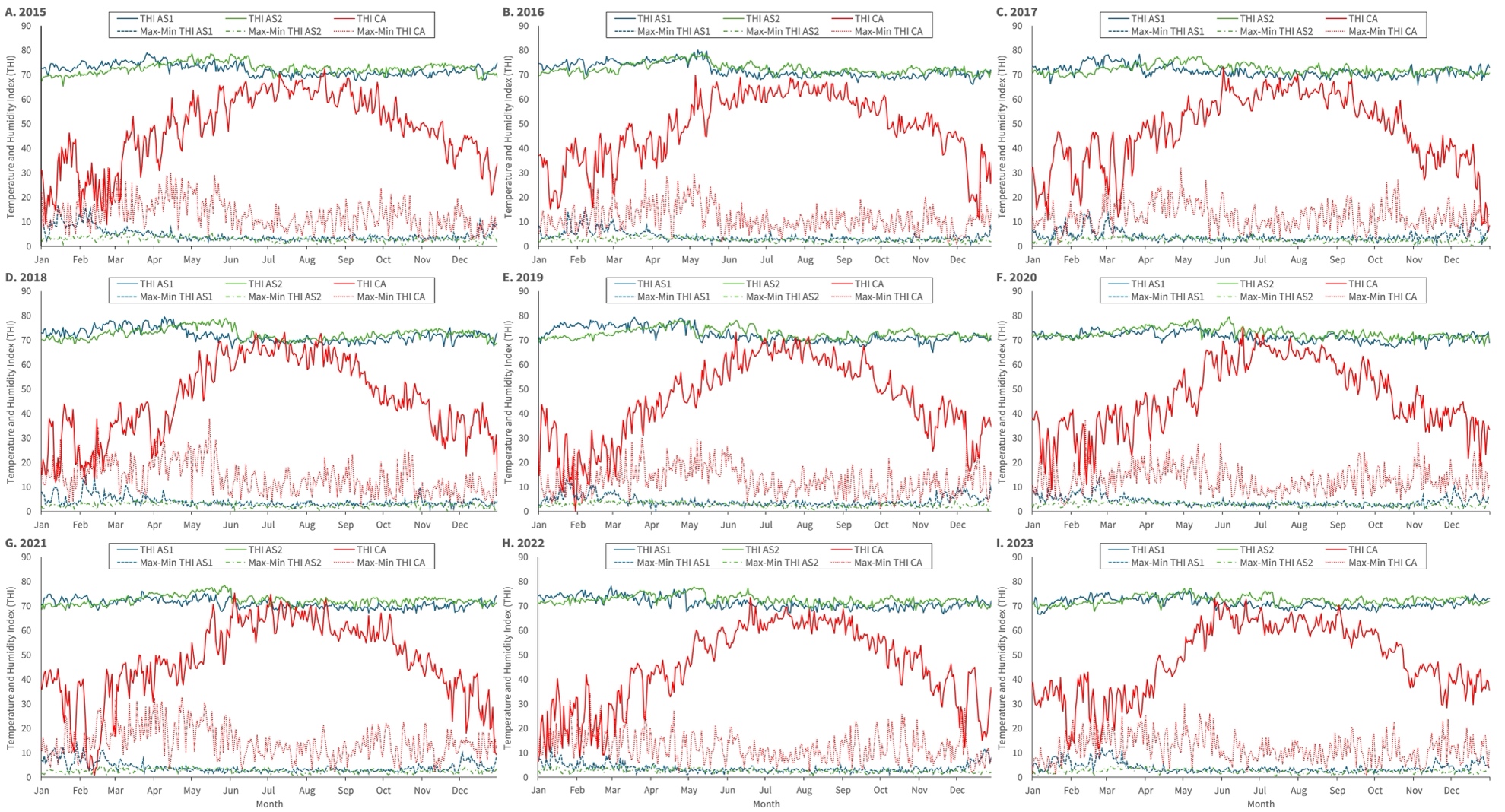


^1^Daily THI range was calculated as the difference between the daily maximum THI and minimum THI values.

^*^Max: Maximum THI; Min: Minimum THI.

Figure S2: Heatmap of estimates (standard errors in parentheses) of genetic (above diagonal) and phenotypic (below diagonal) correlations among analyzed traits: total number born (TNB), number born alive (NBA), number mummified (NM), number stillborn (NSB), farrowing interval (FI), age at first farrowing (AFF) for Southeast Asian herds Landrace (A), Large White (C); for Canadian herds Landrace (B), Large White (D).


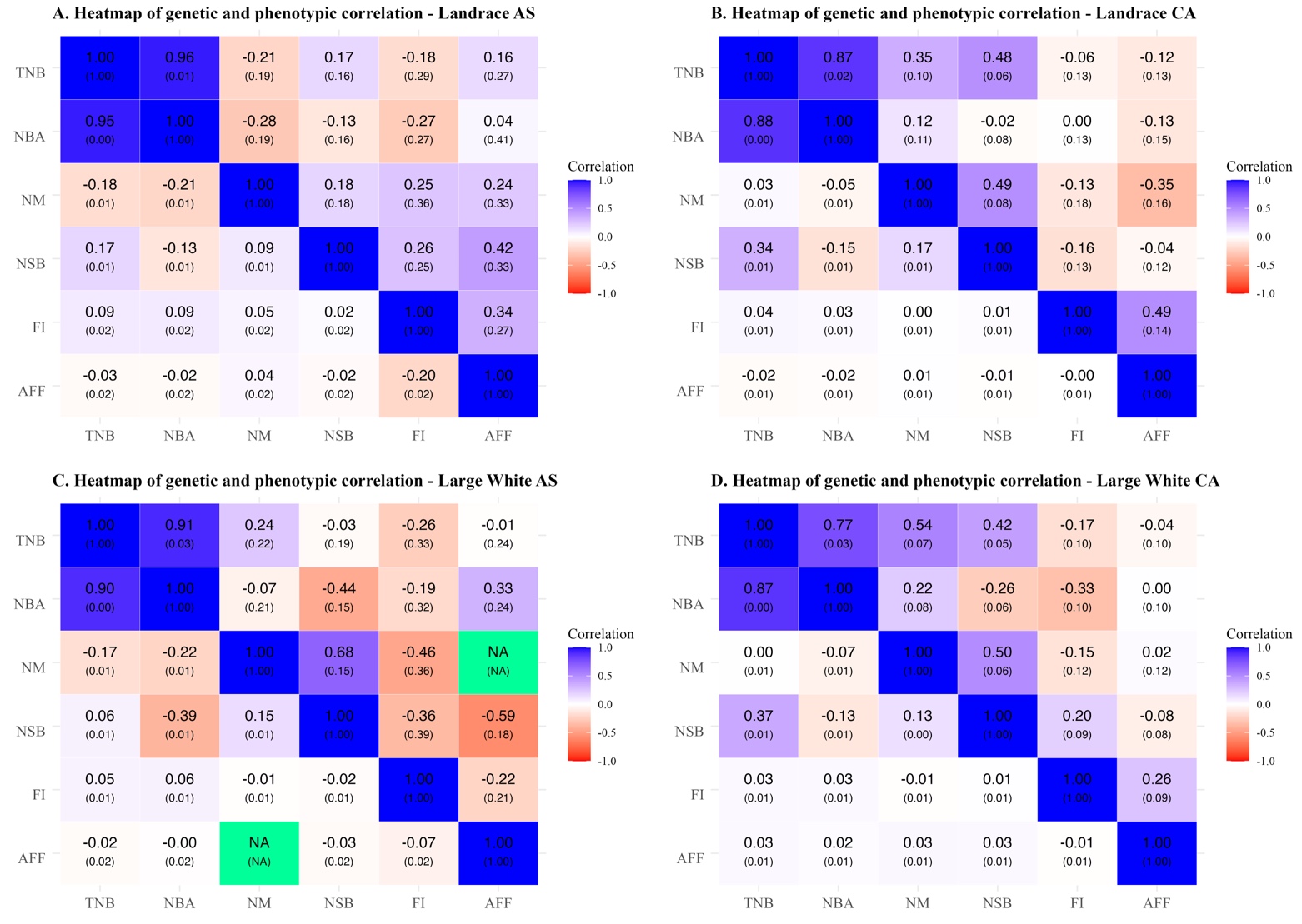


Figure S3: Estimates (and standard error bars) of genetic correlations for reproduction traits between Canada (CA) and each and the combined Southeast Asia herds^1^ (AS1, AS2, AS) for A) Landrace (LR) and B) Large White (LW) non-imported pigs.


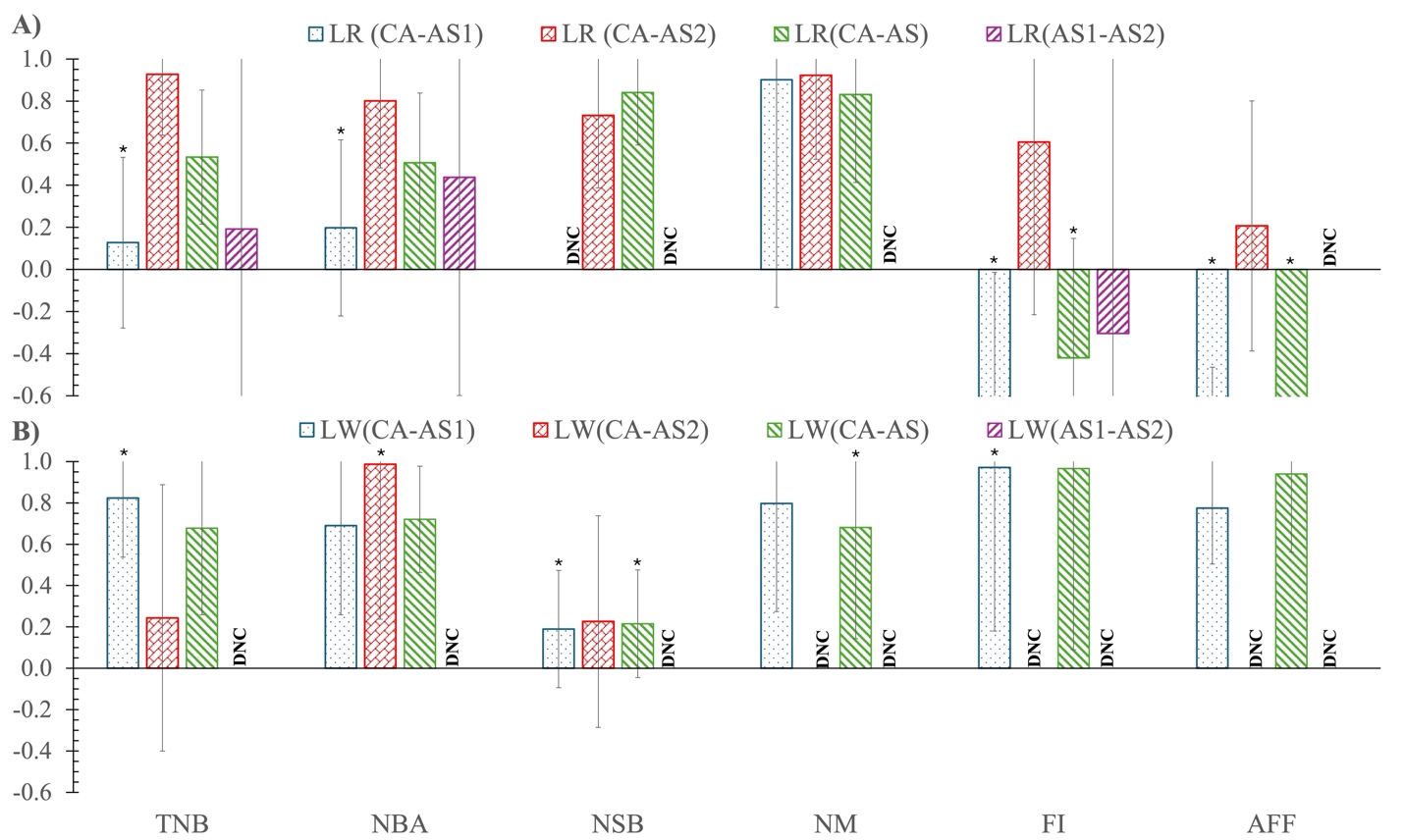


^1^ Genetic correlation between a pair of regions: CA-AS1, Canadian herds and Southeast Asian herd 1; CA-AS2, Canadian herds and Asian herd 2; AS1-AS2, Southeast Asian herd 1 and Southeast Asian herd 2; CA-AS, Canadian herds and two Southeast Asian herds.

*: significantly different from 1 at P < 0.05

DNC: did not converge.

Figure S4: Estimates (and standard error bars) of genetic correlations for reproduction traits between imported and non-imported gilts in each and the combined Southeast Asian herds (AS1, AS2, AS) for A) Landrace (LR) and B) Large White (LW) pigs.


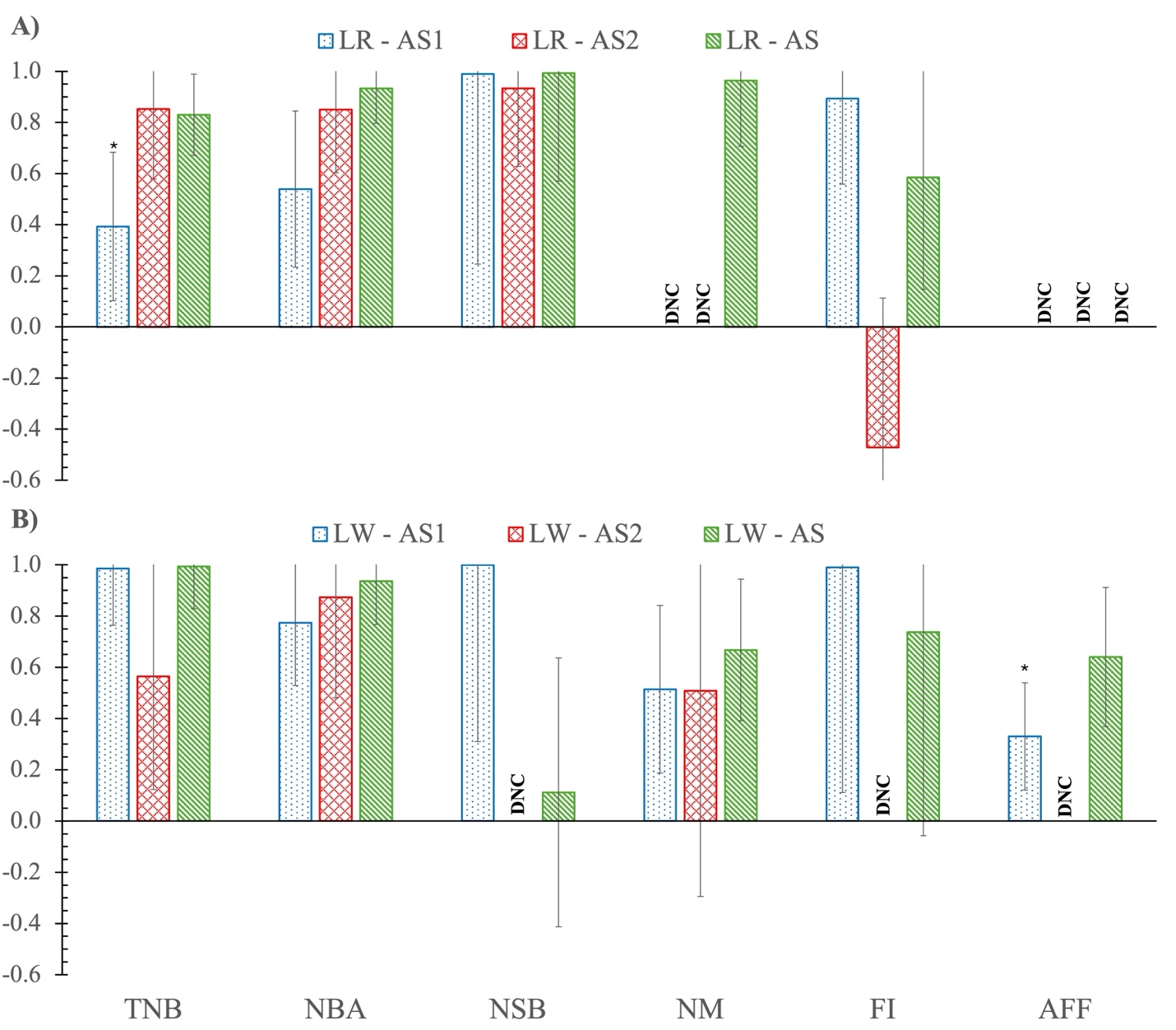


*: significantly different from 1 at P < 0.05

DNC: did not converge.

Figure S5: Estimates (with standard error bars) of genetic correlations for reproductive traits between animals phenotyped during the hot and cold seasons in Canada for Landrace (LR) and Large White (LW) pigs.


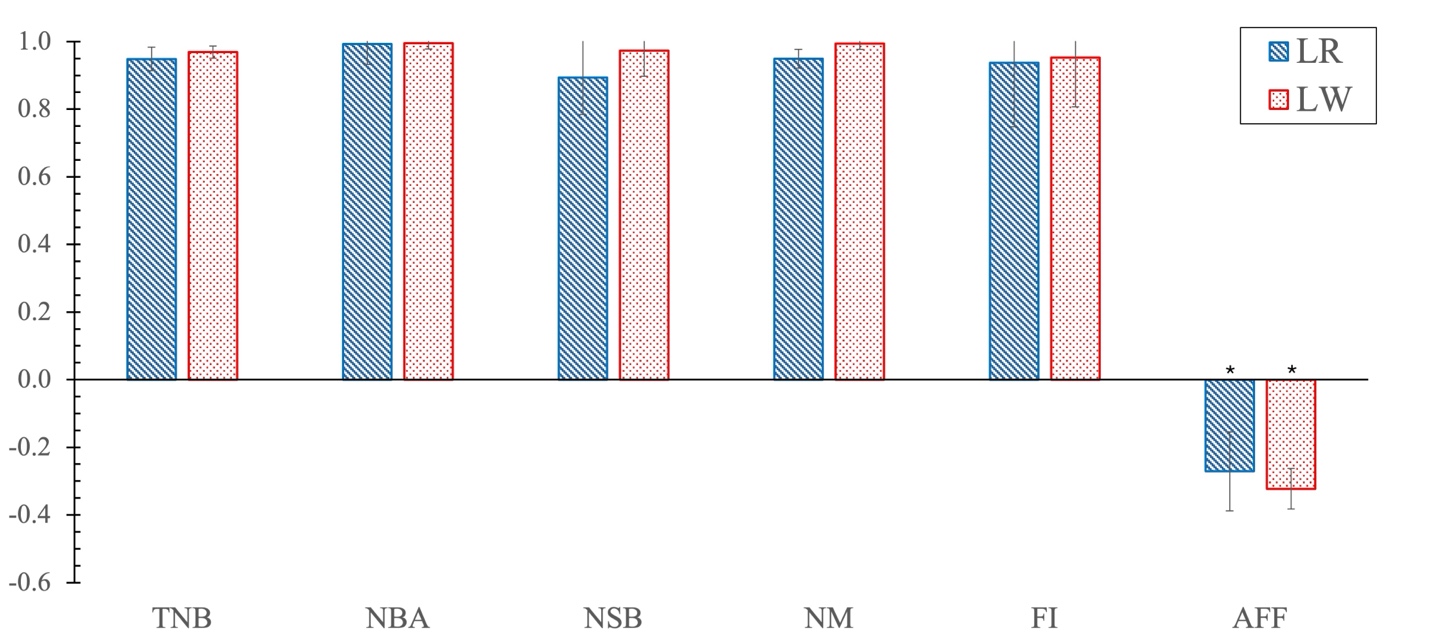


*: significantly different from 1 at P < 0.05

DNC: did not converge.

Figure S6: Estimates (with standard error bars) of genetic correlations^1^ for reproductive traits between animals phenotyped during the hot season in Canada (CA) and each and combined Southeast Asian herds (AS1, AS2, AS) for both Landrace (LR) and Large White LW) pigs.


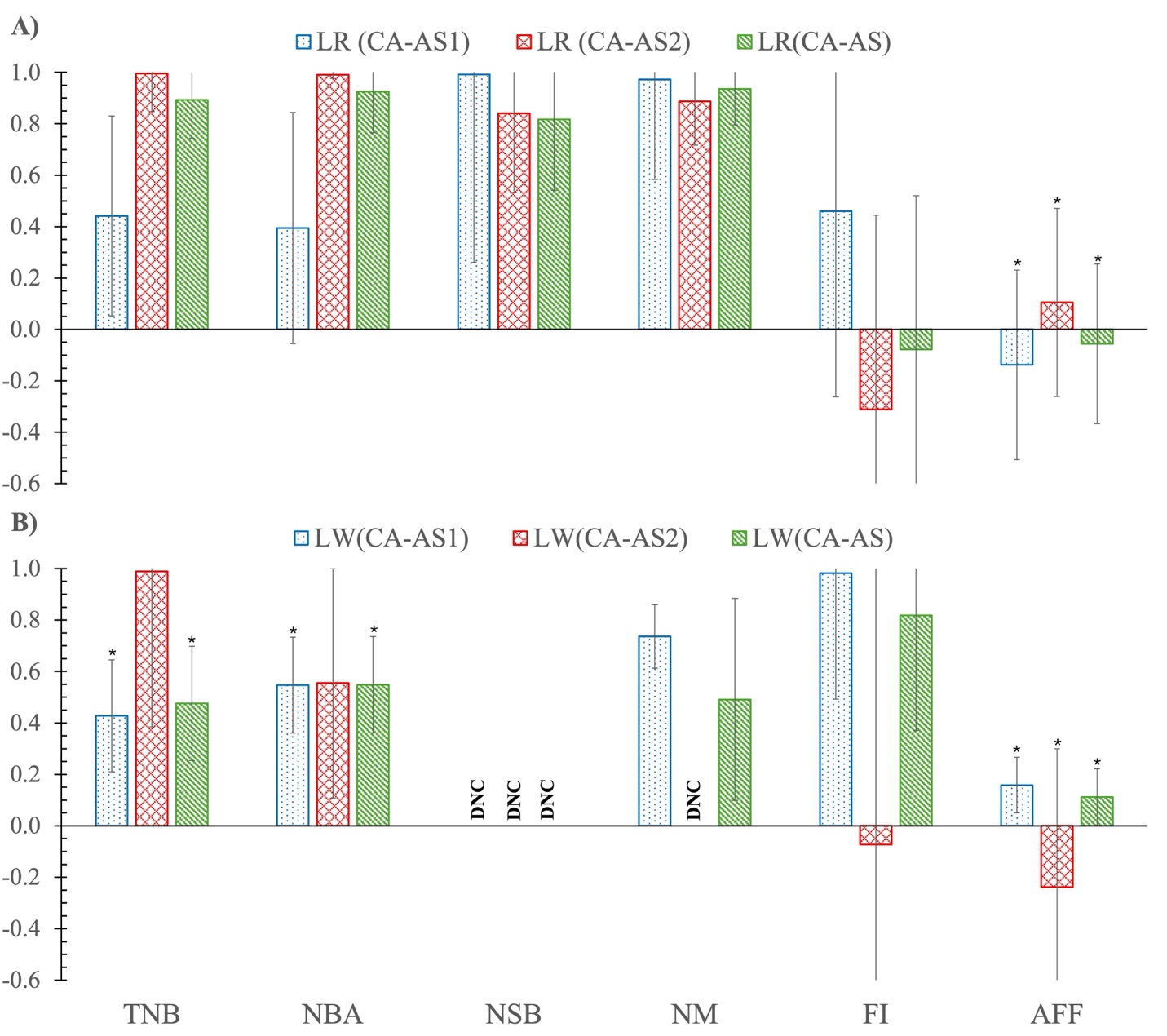


^1^Genetic correlation between a pair of regions: CA-AS1, Canadian herds and Southeast Asian herd 1; CA-AS2, Canadian herds and Asian herd 2; AS1-AS2, Southeast Asian herd 1 and Southeast Asian herd 2; CA-AS, Canadian herds and two Southeast Asian herds

*: significantly different from 1 at P < 0.05

DNC: did not converge.

Figure S7: Estimates (with standard error bars) of genetic correlations^1^ for reproductive traits between animals phenotyped during the cold season in Canada (CA) and each and combined Southeast Asian herds (AS1, AS2, AS) for both Landrace (LR) and Large White LW) pigs.


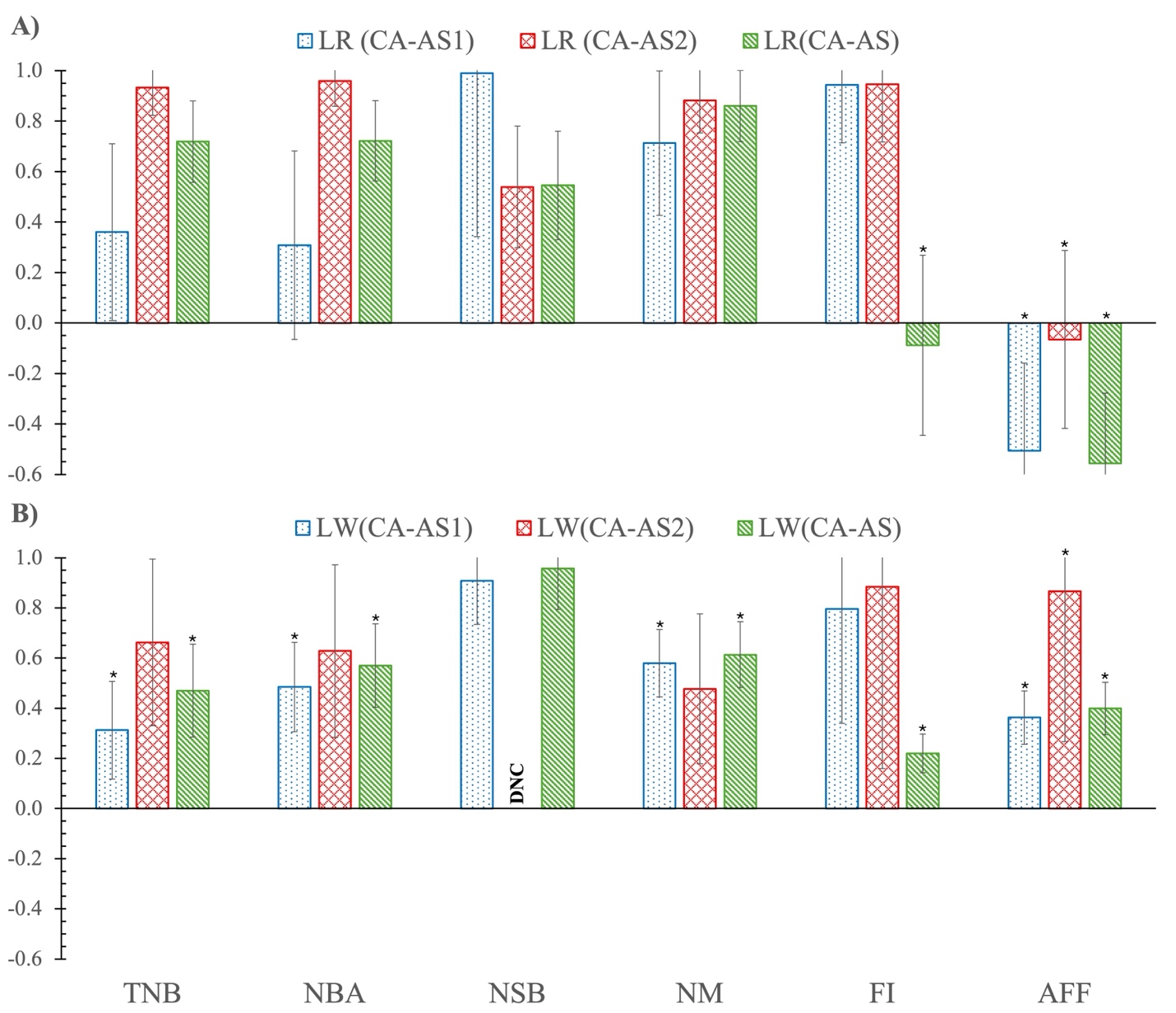


^1^Genetic correlation between a pair of regions: CA-AS1, Canadian herds and Southeast Asian herd 1; CA-AS2, Canadian herds and Asian herd 2; AS1-AS2, Southeast Asian herd 1 and Southeast Asian herd 2; CA-AS, Canadian herds and two Southeast Asian herds

*: significantly different from 1 at P < 0.05

DNC: did not converge.
