# Supplementary figures and images for: Genotype by environment interactions for reproductive performance of North American purebred sows between North America and Southeast Asia
Source: J Anim Sci. 2025 Jun 19;103:skaf191. doi: 10.1093/jas/skaf191 (PMC12272058; doi:10.1093/jas/skaf191)

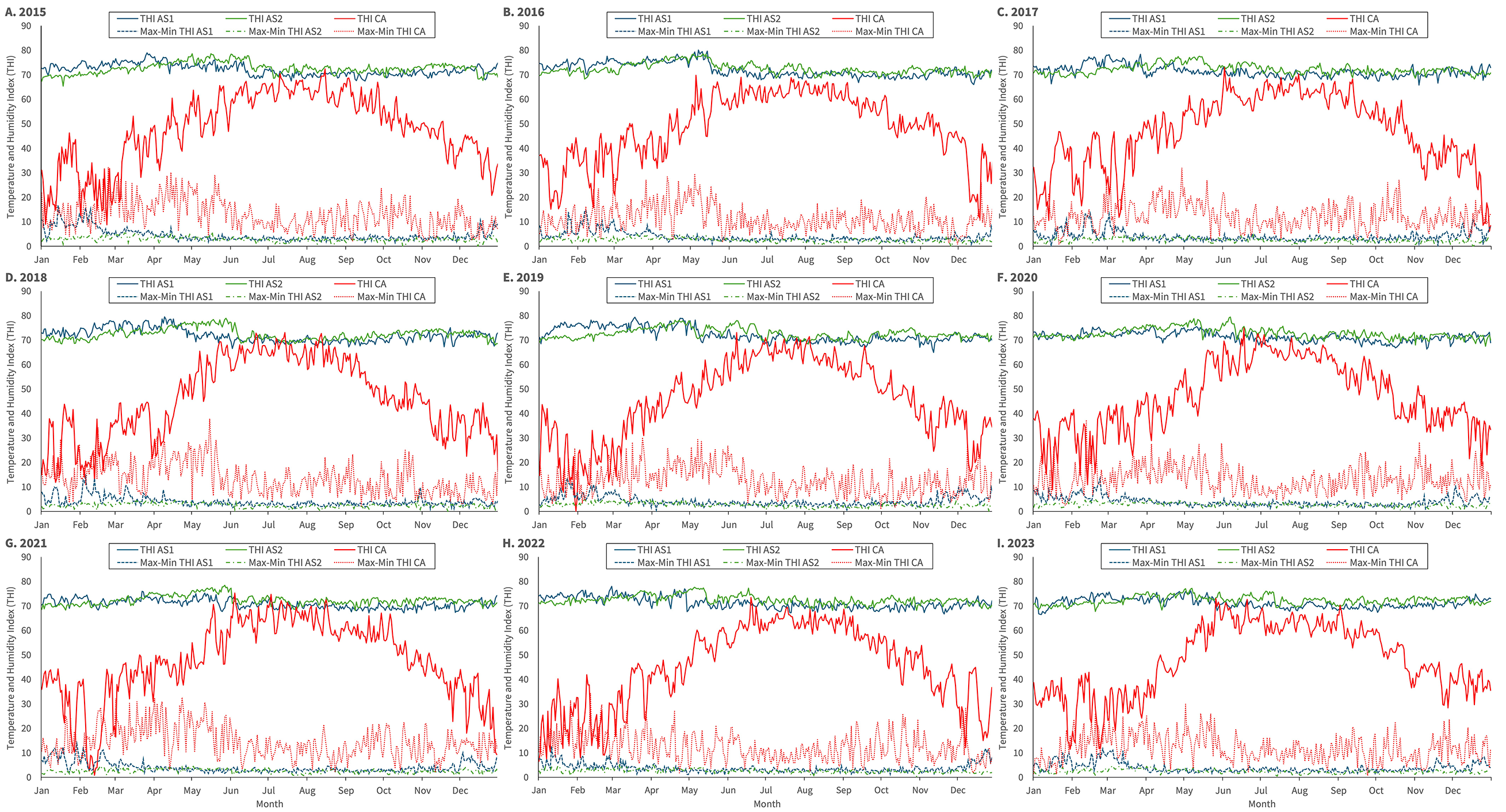

Supplement: skaf191_suppl_Supplementary_Figures_S1-S7_Tables_S1-S3 [file skaf191_suppl_supplementary_figures_s1-s7_tables_s1-s3.zip › Supplementary material/FigureS1.tif]

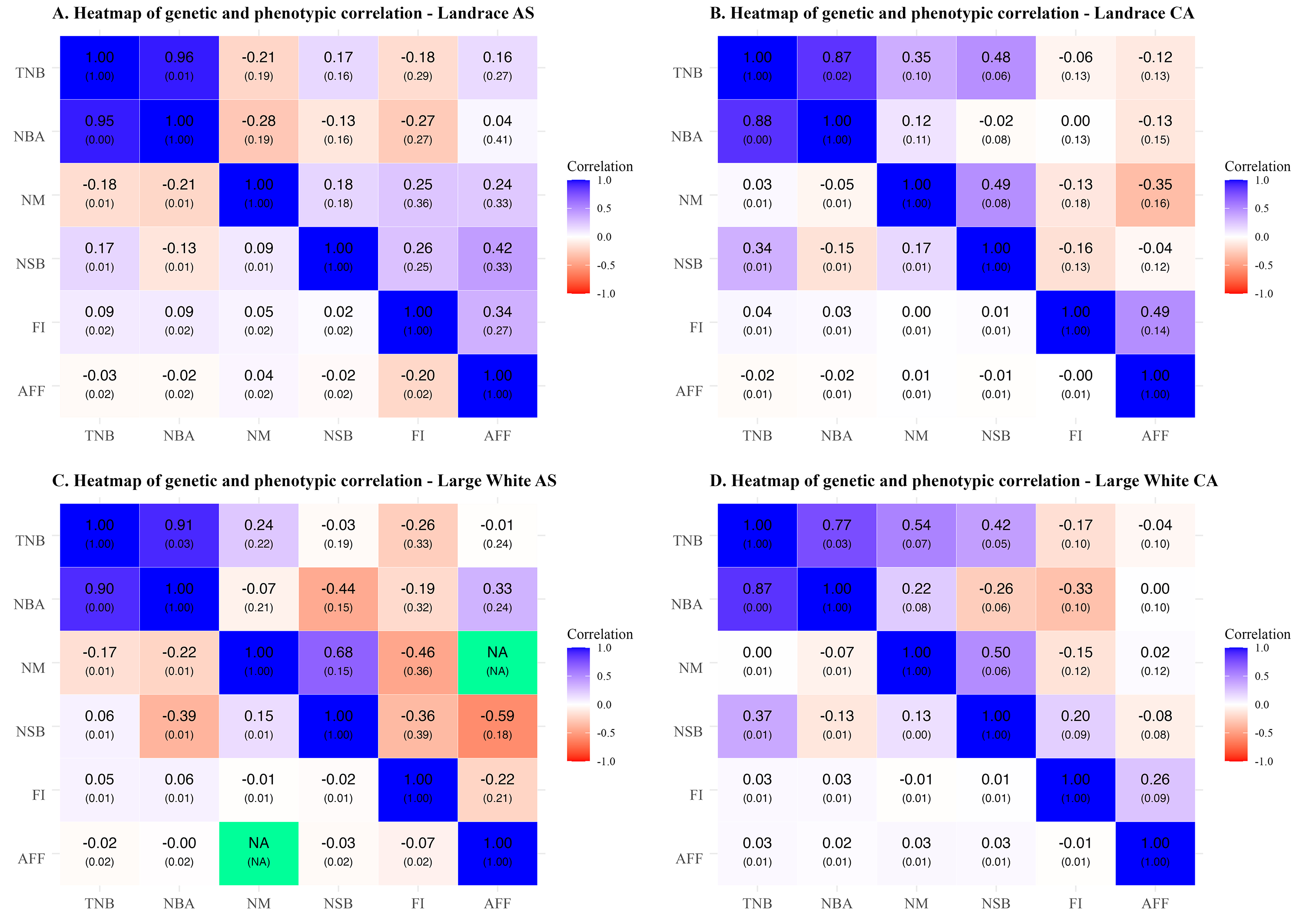

Supplement: skaf191_suppl_Supplementary_Figures_S1-S7_Tables_S1-S3 [file skaf191_suppl_supplementary_figures_s1-s7_tables_s1-s3.zip › Supplementary material/FigureS2.tif]

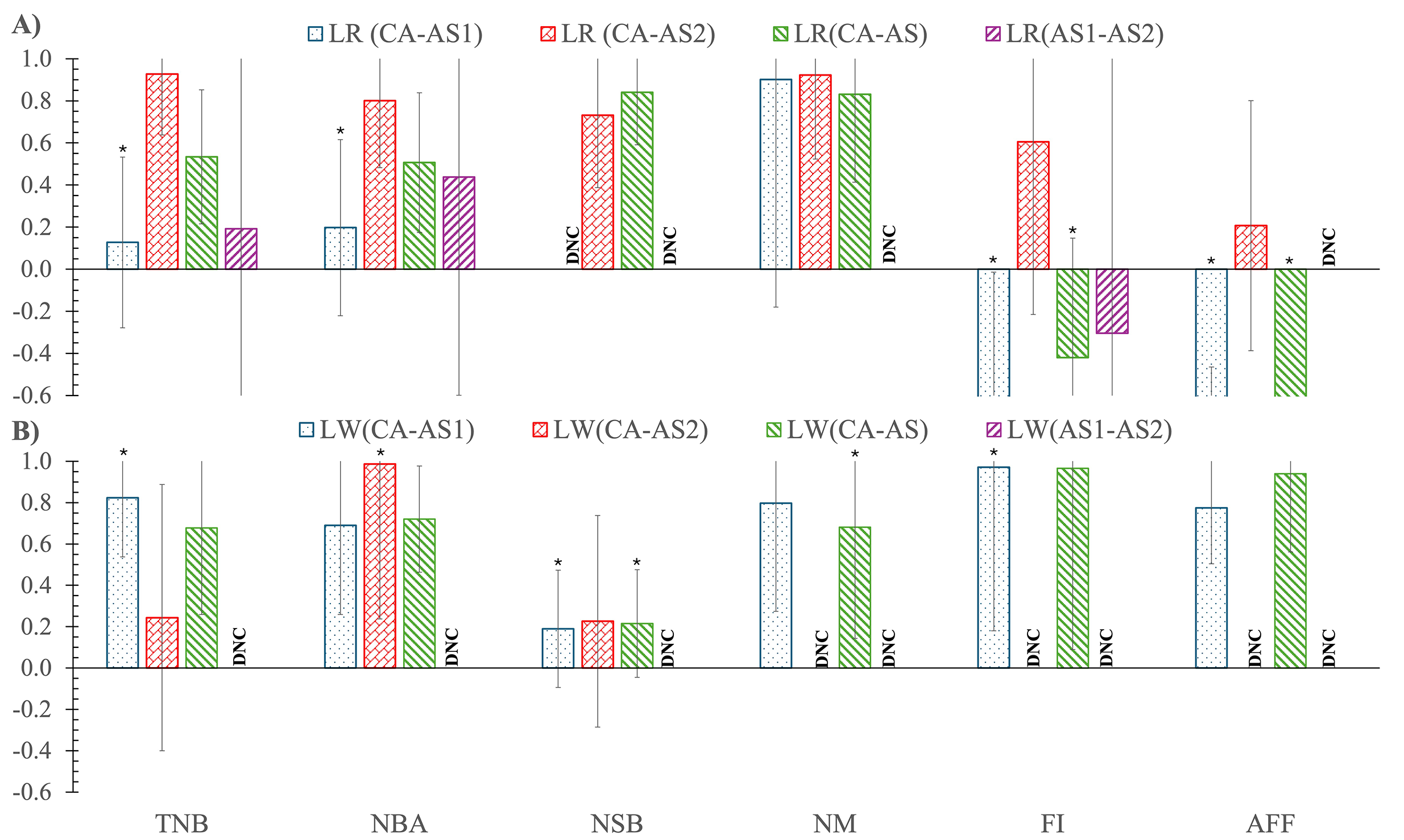

Supplement: skaf191_suppl_Supplementary_Figures_S1-S7_Tables_S1-S3 [file skaf191_suppl_supplementary_figures_s1-s7_tables_s1-s3.zip › Supplementary material/FigureS3.tif]

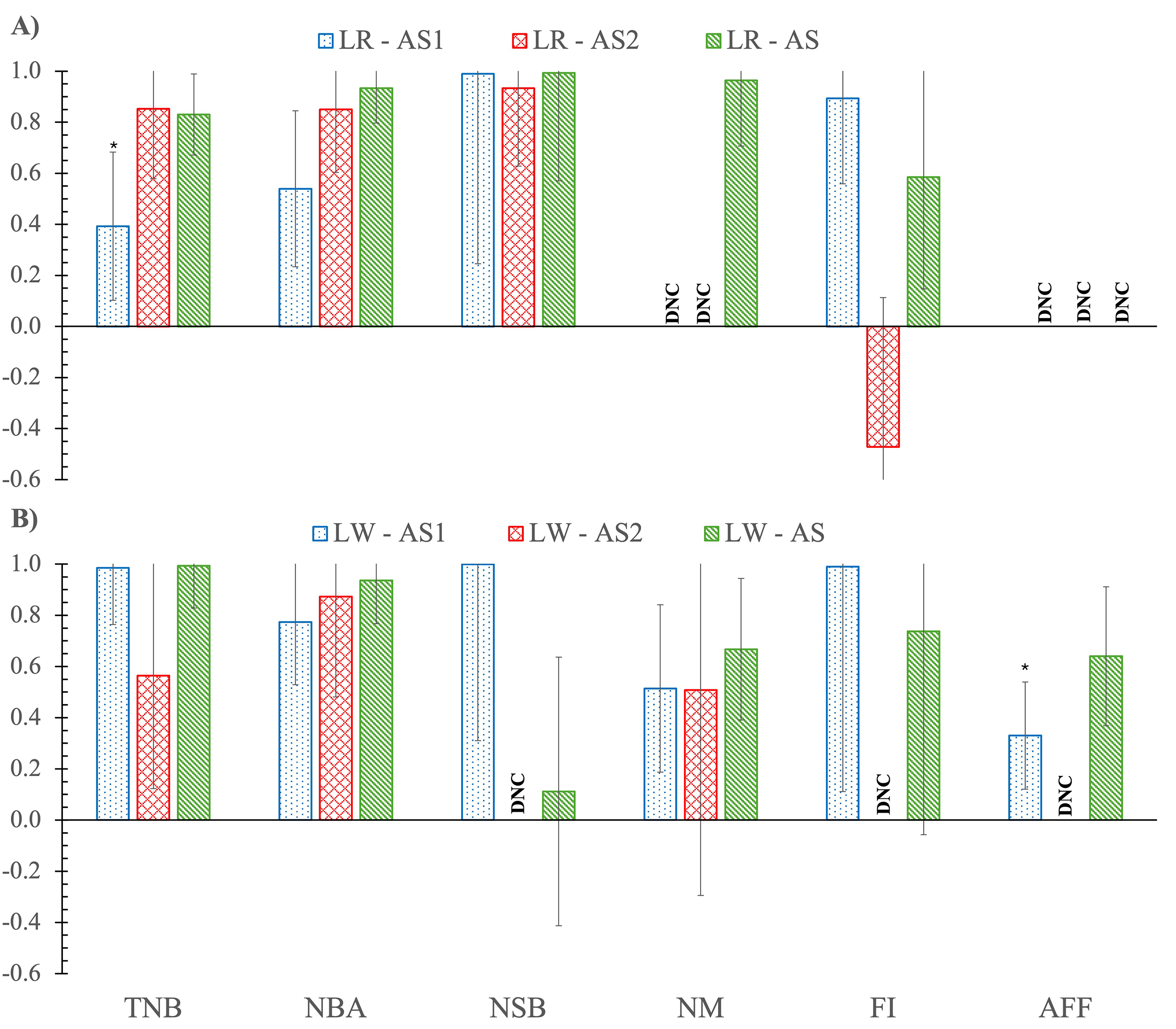

Supplement: skaf191_suppl_Supplementary_Figures_S1-S7_Tables_S1-S3 [file skaf191_suppl_supplementary_figures_s1-s7_tables_s1-s3.zip › Supplementary material/FigureS4.tif]

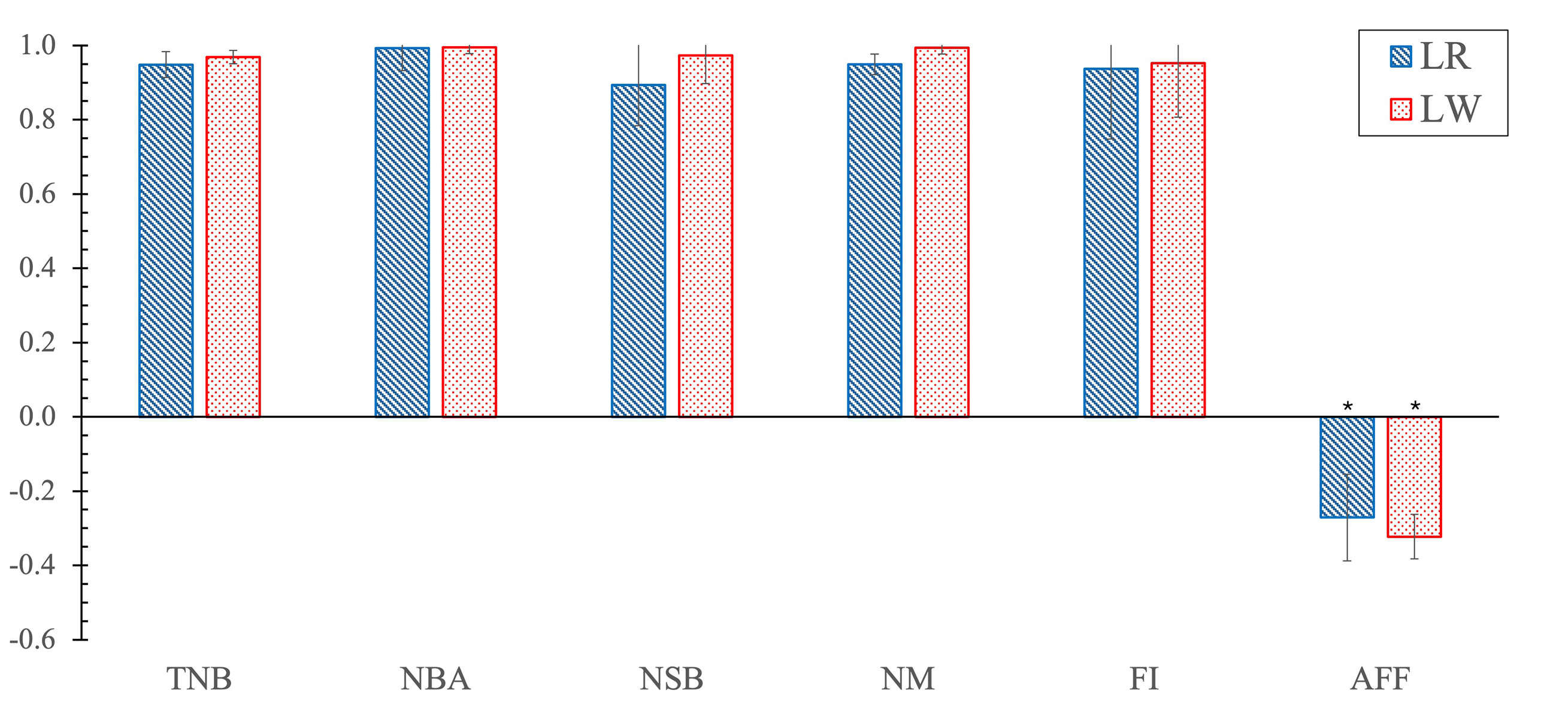

Supplement: skaf191_suppl_Supplementary_Figures_S1-S7_Tables_S1-S3 [file skaf191_suppl_supplementary_figures_s1-s7_tables_s1-s3.zip › Supplementary material/FigureS5.tif]

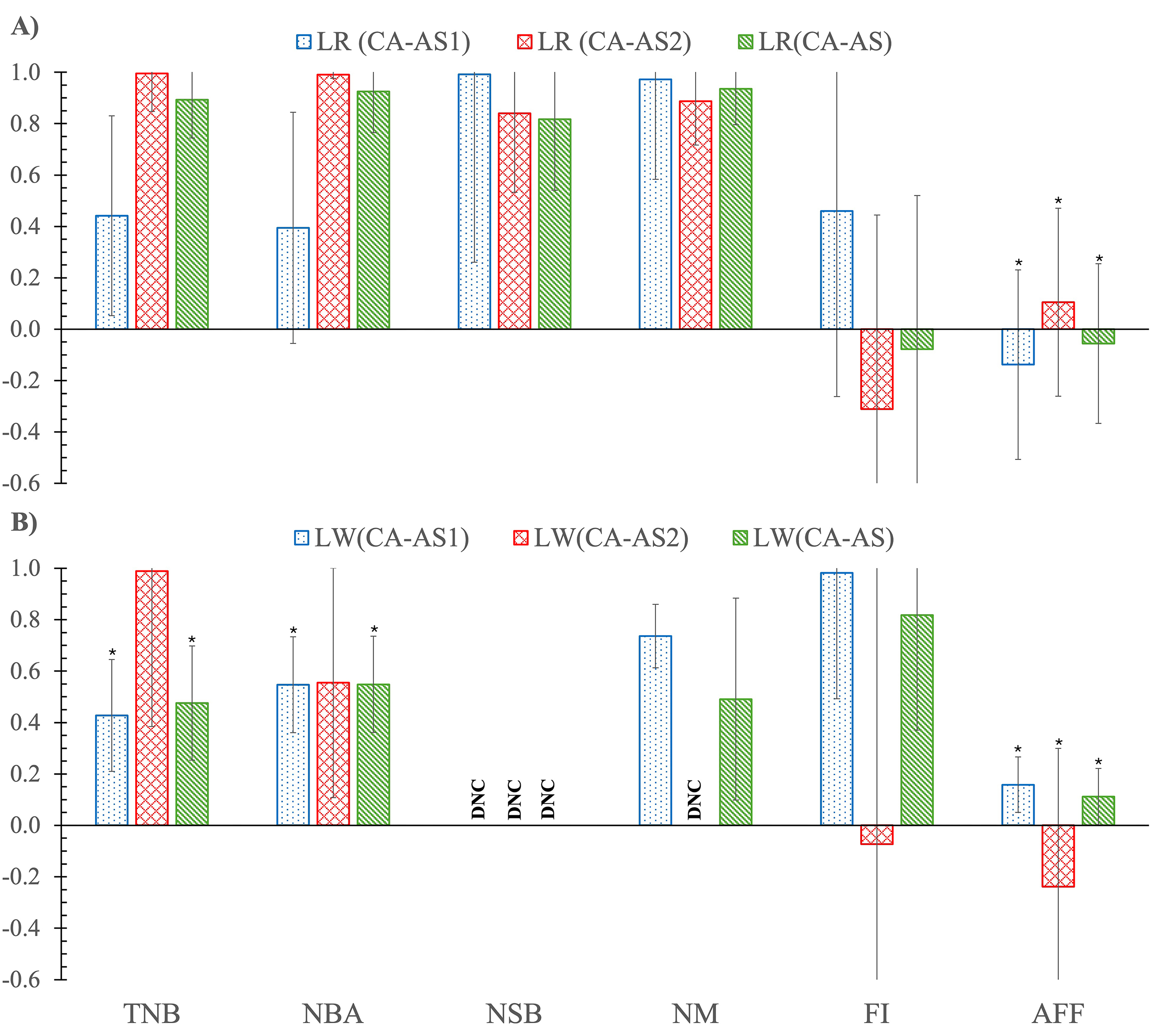

Supplement: skaf191_suppl_Supplementary_Figures_S1-S7_Tables_S1-S3 [file skaf191_suppl_supplementary_figures_s1-s7_tables_s1-s3.zip › Supplementary material/FigureS6.jpg]

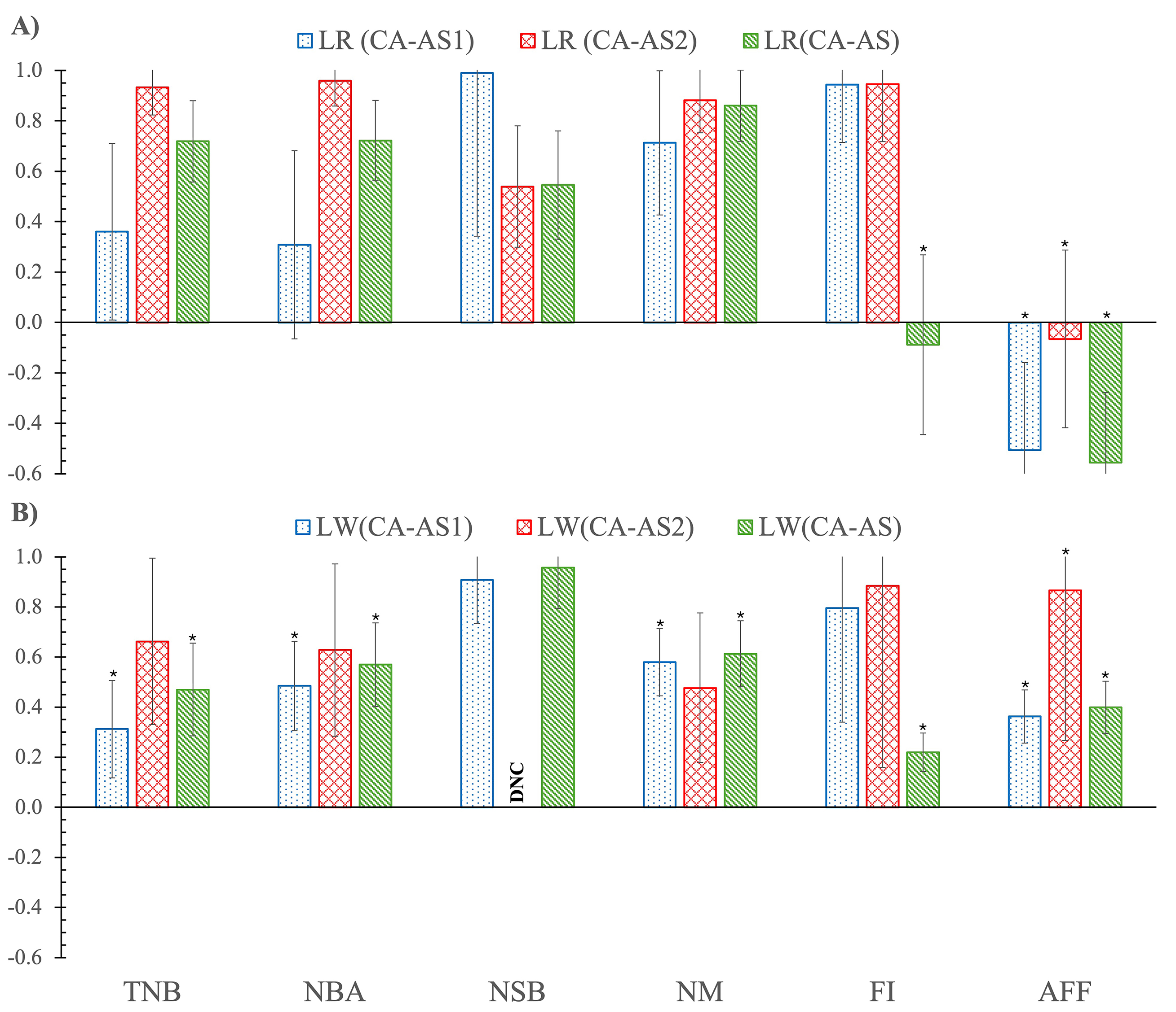

Supplement: skaf191_suppl_Supplementary_Figures_S1-S7_Tables_S1-S3 [file skaf191_suppl_supplementary_figures_s1-s7_tables_s1-s3.zip › Supplementary material/FigureS7.jpg]
